# A droplet-based long-read single-cell epigenome sequencing method for the high-throughput profiling of multidimensional regulatory elements within individual cells

**DOI:** 10.1038/s41421-026-00928-y

**Published:** 2026-10-27

**Authors:** Jiansen Lu, Kexuan Chen, Shengtao Jia, Guodong Chen, Xianhong Yu, Wen Li

**Affiliations:** 1 School of Laboratory Medicine and Biotechnology, Southern Medical University, Guangzhou, Guangdong China; 2 School of Medicine, Jinan University, Guangzhou, Guangdong China; 3 The Second Affiliated Hospital & Liangzhu Laboratory, Zhejiang University School of Medicine, Hangzhou, Zhejiang China; 4 Department of Cardiology, Heart Center, Zhujiang Hospital, Southern Medical University, Guangzhou, Guangdong China; 5 Shanghai MobiDrop Co. Ltd, Shanghai, China

**Keywords:** Chromatin analysis, Histone analysis

## Abstract

Recently, long-read sequencing methods have revolutionized genomic research by allowing the resolution of previously inaccessible regions across the genome. However, the throughput and scalability of these methods have hindered their general application in the field. To address this limitation, we developed droplet-based methods for the single-cell profiling of chromatin accessibility (droplet-based single-cell Nanopore ATAC-seq, dscNanoATAC) and histone modifications (droplet-based single-cell NanoCUT&Tag, dscNanoCUT) by combining a commercial microfluidic device and long-read sequencing platform, achieving a throughput of ~5000 cells in a single experiment and significantly reducing the time and costs compared with previous techniques. With cultured mammalian cells, we demonstrated the ability of dscNanoATAC and dscNanoCUT to detect allele-specific epigenetics, neighboring co-accessibility, and regulatory elements within low-mappability regions. Furthermore, leveraging the haplotype-resolved data generated from dscNanoATAC, we optimized a deep-learning model to predict allele-specific three-dimensional genome structures with high resolution (10 kb). We also applied dscNanoATAC and dscNanoCUT in complex tissues using the mouse brain as an example, and characterized the allele-specific regulation of the imprinting gene *Ube3a* in neurons, obtaining insights into the mechanisms underlying the pathogenesis of Prader-Willi and Angelman syndrome. With dscNanoCUT, we systematically evaluated the activity of transposon elements and revealed their potential regulatory functions in the mouse brain. Together, dscNanoATAC and dscNanoCUT serve as a versatile and scalable long-read epigenome sequencing platform, enabling multidimensional analyses of elements ranging from chromatin accessibility to histone modifications and their interplay between chromatin structures. This platform may advance our understanding of epigenetic regulation in complex biological systems.

## Introduction

Although the primary sequence of the eukaryotic genome remains largely uniform across cells within an organism, remarkably, it allows cells to differentiate into a vast array of distinct types. This extraordinary cellular diversity is primarily underpinned by an intricate spatiotemporal gene regulatory network, consisting of more than one million *cis*-regulatory elements (CREs) embedded within the genome, such as promoters, enhancers, and insulators^1,2^. These elements are established and maintained through the combined interplay of epigenetic modifications and transcription factor events^3,4^. Owing to recent advancements in single-cell epigenomic technologies, such as the assay for transposase-accessible chromatin using sequencing (ATAC-seq)^5^ and cleavage under targets and tagmentation (CUT&Tag)^6^ for histone modification profiling, significant breakthroughs in the precise mapping of epigenetic heterogeneity within individual cells have been made, enabling the comprehensive exploration of the genome-wide epigenetic landscape and increasing our understanding of cell type-specific regulatory mechanisms^7–9^. The emergence of high-throughput, single-cell sequencing methods has further encouraged researchers to investigate complex biological samples at reduced costs and high efficiency. These methods employ combinatorial barcoding strategies such as “split-and-pool”^10,11^ or droplet-based microfluidics platforms^12,13^ to simultaneously capture thousands of cells in one experiment.

However, the majority of existing approaches still rely on short-read sequencing platforms, which exhibit inherent limitations in the accurate resolution of complex genomic regions^14^. Recently, rapid developments in long-read sequencing platforms have revolutionized genomic analysis by enabling a more comprehensive reading of genomic information because of the long read lengths of these platforms. This breakthrough has revealed the “dark matter” within genomes, transcriptomes, and epigenomes, providing new insights into previously inaccessible regions^15^. In particular, the recently developed single-cell NanoATAC-seq (scNanoATAC-seq)^16^ and single-cell NanoSeq-CUT&Tag (scNanoSeq-CUT&Tag)^17^ methods have demonstrated outstanding capabilities in simultaneously detecting epigenetic information (e.g., chromatin accessibility or chromatin modifications) and genetic variations (including structural variants, single-nucleotide variants, and copy number variations) within individual cells. Owing to their long sequencing reads, these methods can identify allele-specific peaks, assess neighboring co-accessibility/co-occupation, and profile the chromatin modifications in individual copies of repetitive elements with high efficiency. However, current single-cell sequencing technologies based on long-read sequencing platforms are all plate-based, which significantly limits their widespread application because of their high costs, low throughput, and labor-intensive workflows. Additionally, a critical gap remains in our understanding of the precise interactions within the three-dimensional (3D) genome. Although long-read Hi-C technologies, such as scNanoHi-C^18^, have enabled haplotype-specific Hi-C analyses, their resolution remains limited, making in-depth investigations into allele-specific chromatin interactions and their functional implications difficult.

To address these challenges, we present droplet-based single-cell Nanopore ATAC-seq (dscNanoATAC) and droplet-based single-cell NanoCUT&Tag (dscNanoCUT), which leverage Nanopore long-read sequencing and involve a commercial microfluidic device for the high-throughput, genome-wide profiling of the epigenome at the single-cell level. We also developed a computational pipeline tailored for long-read droplet sequencing data to investigate allele-specific epigenetics, co-accessibility, and regulation in complex genomic regions. Leveraging the high efficiency of dscNanoATAC in haplotype tagging, we developed an allele-specific C.Origami workflow that predicts allele-specific chromatin interaction maps at high resolution (10 kb). We further demonstrated the utility of dscNanoATAC and dscNanoCUT by applying them to the mouse brain and demonstrated their ability to dissect information from complex primary tissues. Collectively, our methods provide scalable and versatile tools that could lead to a comprehensive understanding of epigenetic regulatory elements in complex tissues.

## Results

### Technology workflow and data quality

**Fig. 1:**
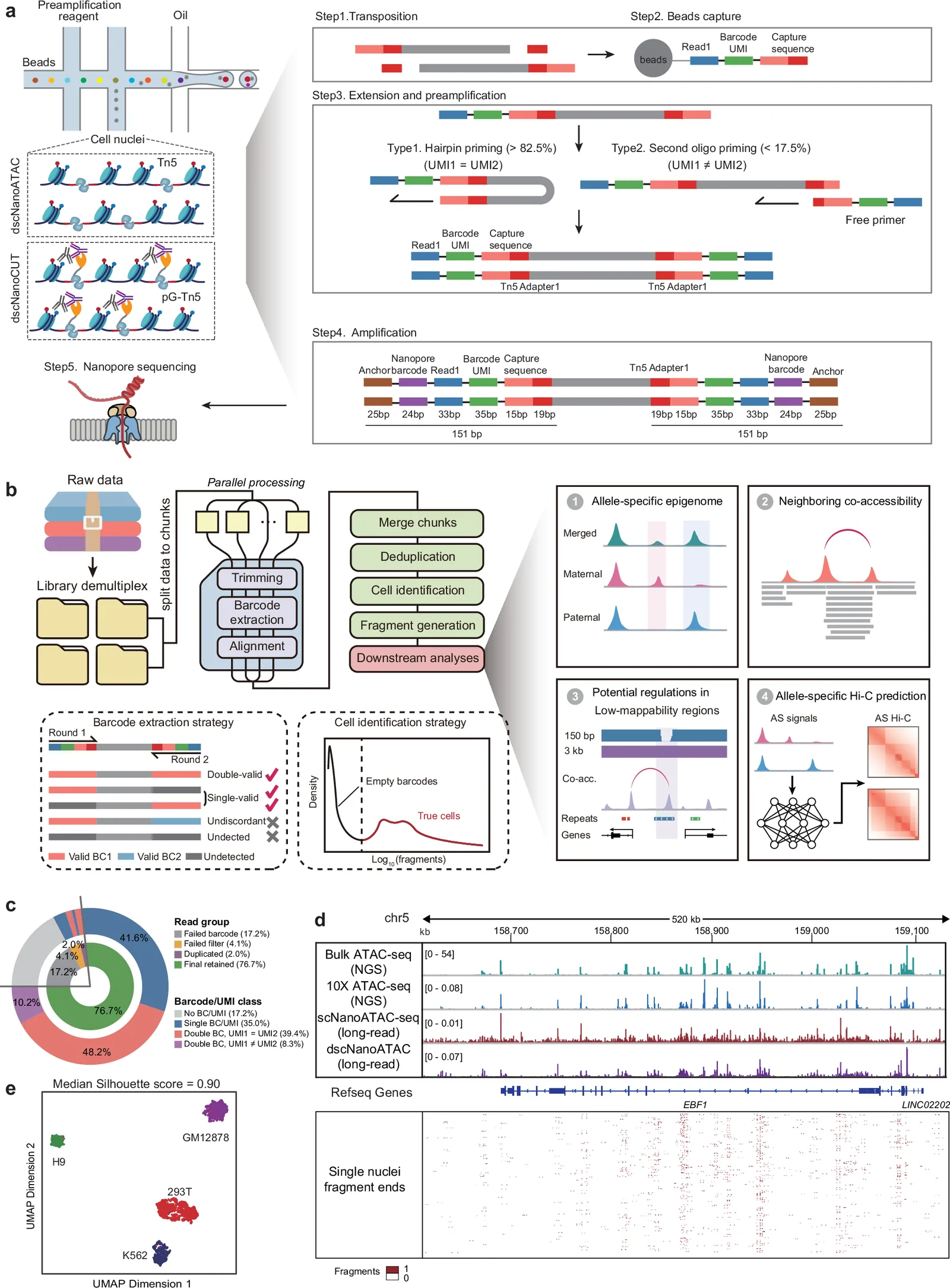
Design of a droplet-based single-cell epigenome method using the Nanopore platform. **a** Schematic illustrating the experimental principles and workflow of dscNanoATAC and dscNanoCUT. **b** Schematic workflow illustrating the computational pipeline for preprocessing and analyzing dscNanoATAC and dscNanoCUT data. **c** Summary of the read groups and barcode/UMI classes during the preprocessing steps of the GM12878 dscNanoATAC data. The inner ring shows the read groups, and the outer ring stratifies the reads on the basis of their barcode and UMI classes. The percentage of the barcode/UMI class in the final retained reads is displayed on the outer ring. **d** Genome browser view showing a representative region of the pseudo-bulk and binarized single-cell chromatin accessibility signals of dscNanoATAC in GM12878, along with bulk ATAC-seq (short-read-based), 10x ATAC-seq (short-read-based)^20^, and scNanoATAC-seq datasets (long-read sequencing-based)^16^. The represented gene, *EBF1*, encodes a B-cell-specific transcription factor. **e** UMAP of four cell lines following the use of dscNanoATAC. The silhouette coefficient is calculated on the basis of the top 2 to 5 latent semantic indexing (LSI) dimensions.

As illustrated in Fig. 1a, we developed a droplet microfluidics-based ATAC-seq method, termed dscNanoATAC, for single-cell chromatin accessibility profiling on the Nanopore long-read sequencing platform (Fig. 1a; Supplementary Fig. S1a). Briefly, nuclei are first subjected to Tn5 transposition at accessible chromatin regions^7^. A key design feature of dscNanoATAC is the use of two identical Tn5 adapters, each engineered to be compatible with oligonucleotide sequences on microfluidic beads. We also developed dscNanoCUT, in which nuclei are incubated with an antibody that targets a specific protein, followed by CUT&Tag using protein G-fused Tn5 (pG-Tn5) transposase (Fig. 1a; Supplementary Fig. S1b). After microfluidic encapsulation, cell identity is conferred through the incorporation of DNA barcodes, after which the bead-captured molecules may transiently form a hairpin structure through complementarity between the two Tn5 adapters, enabling 3’ adapter extension and ultimately resulting in both ends of the fragment harboring identical barcode and unique molecular identifier (UMI) sequences. Alternatively, a second primer may be involved in the 3’ priming, which results in two different UMIs. These two mechanisms account for 82.5% and 17.5%, respectively, of the dual-barcode reads (Supplementary Fig. S2). To encourage the generation of long DNA fragments, the preamplification extension time was lengthened. Finally, a single amplification primer with 24-bp Nanopore barcodes was used for the final amplification step. The resulting amplicons were ~4 kb in length and were appropriate for sequencing on the Nanopore platform (Supplementary Figs. S1c, d, S2). This droplet-based approach significantly reduces hands-on time and enhances efficiency, particularly in cell barcoding and library preparation processes, offering a streamlined and scalable workflow for high-throughput applications. For example, the library construction time for ~5000 cells was reduced from at least one week with the plate-based scNanoATAC to just one day with dscNanoATAC (Supplementary Fig. S3a, b). In terms of cost, on the basis of current prices, the experimental cost was reduced by more than fivefold, with key reagent costs decreasing from ~$3915 to ~$745 while the total cost including sequencing decreased from ~$9915 ($1.98/cell) to ~$1945 ($0.39/cell) (Supplementary Fig. S3c). In other words, with the same reagent budget, compared with the plate-based method, dscNanoATAC can process five times the number of single cells. To facilitate data analysis, we also developed a scalable and reproducible pipeline. Like in previous methods, only the ends of long fragments represent the ATAC or CUT&Tag signal^16,17^. Therefore, we implemented a stringent filtration step to ensure the accurate identification of Tn5 insertion sites (see Materials and Methods). One advantage of our method is that the majority of the generated reads possess paired-end cell barcodes via “hairpin priming”, which can be used for mutual correction and to mitigate the impact of sequencing errors, as well as the implementation of a local minima-based strategy as previously described to distinguish true cells from empty droplets^19^ (Fig. 1b).

To assess the feasibility of our droplet-based, long-read single-cell epigenome sequencing methods, we first applied dscNanoATAC and dscNanoCUT to human K562 and mouse NIH3T3 fibroblast lines with different cell loading for species-mixing experiments. The observed collision rates were 0.85% (low loading, dscNanoCUT) and 6.48% (high loading, dscNanoATAC), respectively, demonstrating minimal cross-contamination among individual cells in both the dscNanoCUT and dscNanoATAC experiments (Supplementary Fig. S3d, e). As a proof of concept, we then applied dscNanoATAC to individual cells from GM12878, a female human lymphoblastoid cell line (LCL) whose chromatin accessibility and histone modification landscapes have been extensively characterized. A total of 66 Gb of data were generated with inputs of 10,000 cell nuclei, which showed high demultiplexing efficiency (82.8% of the reads were assigned to valid cell barcodes; Supplementary Fig. S4a and Table S1). After preprocessing, the overall efficiency (the proportion of valid reads to all sequencing reads) in dscNanoATAC was ~76.7% (including 41.6% single barcodes, 48.2% dual barcodes with equal UMIs, and 10.2% dual barcodes with unequal UMIs; Fig. 1c), and 8057 cells passed the quality filter (requiring > 500 unique fragments or mapped genomic inserts). Analysis of aggregated chromatin accessibility across single cells with dscNanoATAC and other published approaches (including the long-read sequencing-based scNanoATAC-seq^16^ and the short-read sequencing-based 10x scATAC-seq^20^) revealed high correlations among the approaches and a high signal-to-noise ratio for dscNanoATAC (Fig. 1d; Supplementary Fig. S4b). Correlation analysis of genome-wide chromatin accessibility between independent replicates confirmed the high reproducibility of the results of dscNanoATAC (*R* = 0.83) (Supplementary Fig. S4c).

We also performed systematic benchmarking analyses between dscNanoATAC and other methods to evaluate the performance of our proposed method. At a relatively low sequencing depth (less than 0.01 Gb/cell), compared with scNanoATAC-seq (~0.1 Gb/cell), dscNanoATAC exhibited similar transcription start site (TSS) enrichment scores, promoter ratio, and mitochondrial ratio. While the read length of dscNanoATAC (~3 kb) is shorter than that of scNanoATAC-seq (~5 kb), it still significantly exceeds that of short-read-based methods. Moreover, the fraction of reads in peaks (FRiP) of dscNanoATAC was greater than that of scNanoATAC-seq, which also reflects the high signal-to-noise ratio of the former (Supplementary Fig. S4d–g). In addition, we analyzed the genomic distribution of accessible peaks identified with 10x scATAC-seq, scNanoATAC-seq, and our dscNanoATAC method in GM12878 cells using annotations derived from ENCODE candidate CREs (cCREs) and RefSeq genes and showed consistent results across the datasets (Supplementary Fig. S4h–k). Overall, these results indicated that dscNanoATAC reliably detected chromatin accessibility at the single-cell level.

### dscNanoATAC accurately distinguishes different cell types

An important application of single-cell techniques is the differentiation of cell types within a heterogeneous population. Therefore, we applied dscNanoATAC to mixtures of four cultured human cell lines (GM12878, the embryonic kidney cell line HEK293T, the chronic myelogenous leukemia cell line K562, and the T lymphocyte cell line H9) to evaluate its ability to distinguish different cell types. After these four cell lines were mixed, dscNanoATAC was applied to one reaction using a microfluidic device. After filtering, 7064 single cells — 2827 HEK293T cells, 1986 GM12878 cells, 1237 K562 cells, and 996 H9 cells — were retained, exhibiting a median TSS enrichment score of 2.2 and an average of 1918 unique fragments per cell (Supplementary Fig. S5a). Despite the relatively shallow sequencing depth, the cell line clusters were clearly separated after uniform manifold approximation and projection (UMAP) dimensionality reduction, with a silhouette coefficient of 0.90 (Fig. 1e). We also showed that activation of canonical marker genes and the footprints of cell type-specific transcription factors, such as GATA in the K562 line and PAX5 in the GM12878 line, were faithfully detected in our data (Supplementary Fig. S5b–d).

### dscNanoATAC efficiently identifies allele-specific chromatin accessibility and neighboring co-accessibility events

**Fig. 2:**
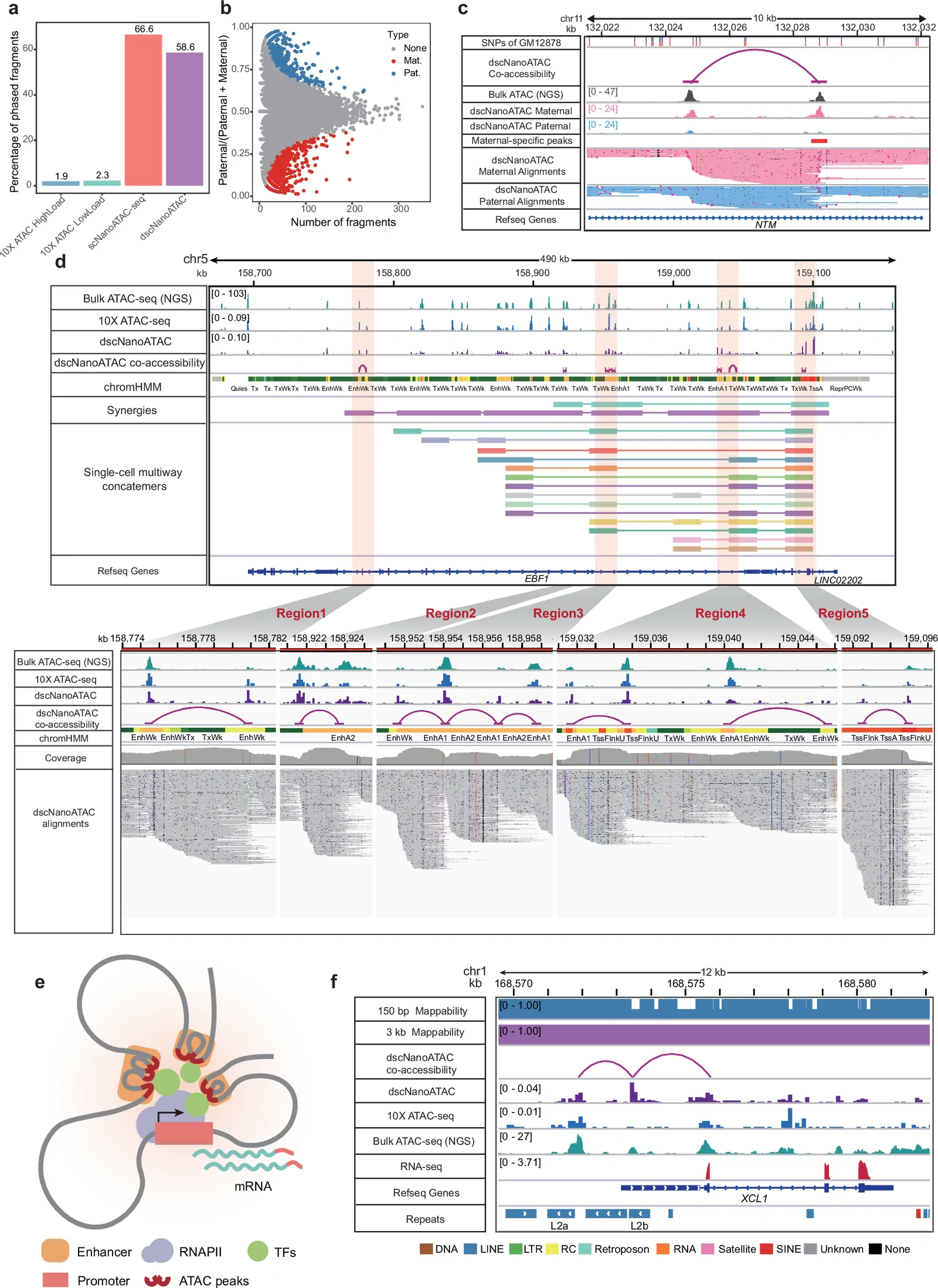
dscNanoATAC is a versatile tool for epigenome profiling. **a** Proportion of haplotagged fragments with dscNanoATAC, and high-loading and low-loading 10x scATAC-seq and scNanoATAC-seq. **b** The ratio between paternal fragments and the total number of haplotyped reads for each peak in GM12878 identified by dscNanoATAC. Statistically significant ASPs (FDR < 0.05) are highlighted in blue (paternal) and red (maternal). **c** Genome browser view showing an example of ASPs within the maternally imprinted *NTM* locus in the GM12878 cell line, as well as the co-accessibility between the two peaks. **d** Genome browser view showing multiple chromatin co-accessibility events at the *EBF1* locus, which are consistent with the synergies and multiway concatemers (high-order promoter–enhancer interactions) predicted by scNanoHi-C. The red shadow highlights the co-accessibility region inside *EBF1*. ChromHMM annotations: TssA, active TSS; TSSFlnk, flanking TSS; TSSFlnkU, flanking TSS upstream; Tx, strong transcription; TxWk, weak transcription; EnhA1, active enhancer 1; EnhA2, active enhancer 2; EnhWk, weak enhancer; ReprPCWk, weak repressed Polycomb; Quies, quiescent/low. **e** Schematic diagrams illustrating the co-accessible peaks embedded in multiple enhancers that simultaneously interact with the same promoter to ensure its high expression. **f** Genome browser view revealing accessible signals at LINE elements upstream of *XCL1*, which also exhibit co-accessibility with the *XCL1* promoter.

The relatively long read length of dscNanoATAC offers a distinct advantage in haplotagging^21^ because of its high capture rate of heterozygous single-nucleotide polymorphisms (SNPs). Indeed, when GM12878 cells were used, the percentage of reads that could be directly haplotagged with dscNanoATAC (58.6%) was comparable to that with scNanoATAC-seq (66.6%) and close to 30-fold that with the 10x scATAC-seq method (~2.0%) (Fig. 2a). We next identified allele-specific peaks (ASPs) using dscNanoATAC data and reported that ~2.2% (1180/53,789) of the total peaks were allele specific. Similar to scNanoATAC-seq^16^, dscNanoATAC also detected X chromosome inactivation events; to this end, we found that the frequency of ASPs on the X chromosome was notably greater than that on autosomes, with a predominant bias toward maternal alleles, which is consistent with the paternal X chromosome inactivation reported in the GM12878 cell line (Fig. 2b; Supplementary Fig. S6a). Several ASPs located in known genomic imprinting loci were also captured, such as those including the maternally expressed gene *NTM* and the paternally expressed gene *SGCE*^22^ (Fig. 2c; Supplementary Fig. S6b and Table S2). These results validated the ability of dscNanoATAC to highly efficiently identify ASPs.

Co-accessibility analysis allows the inference of potential enhancer–promoter interactions, which facilitates the reconstruction of gene regulatory networks. In short-read-based scATAC-seq, co-accessibility is typically defined by peak correlations across single cells^23^. In contrast, the long reads generated by dscNanoATAC allow us to identify physical links between nearby peaks, yielding a more direct measure of co-accessibility (Supplementary Fig. S6c). To identify such neighboring co-accessibility events, we adapted previous methods related to scNanoATAC-seq^16^ and identified 9374 co-accessibility events in GM12878 cells (Supplementary Fig. S6d and Table S3).

We next compared our physical-linkage-based approach with the correlation-based Cicero method^23^. All neighboring co-accessibility events identified by our method were also detected by Cicero. Moreover, events captured by both methods exhibited significantly higher Cicero scores, indicating that our neighboring co-accessibilities represent the most significant correlation-based events (Supplementary Fig. S6e). For example, we identified multiple co-accessibility events at the *EBF1* gene locus, which encodes a B-cell-specific transcription factor essential for LCL proliferation and survival^24^. These sites precisely overlap with the sites of previously reported higher-order promoter–enhancer interactions identified by scNanoHi-C^18^, suggesting that co-accessible regions are spatially proximal and organized into hierarchical structures for promoting target gene expression (Fig. 2d, e). We consistently observed similar patterns for the EBV super-enhancer-associated gene *MIR155HG* and the B-cell-specific transcription factor *IKZF2* (Supplementary Fig. S7).

These findings demonstrate that chromatin co-accessibility analysis can complement higher-order chromatin interaction analysis at a relatively high resolution, providing a more comprehensive evaluation of regulatory interactions among multiple enhancers and promoters. Furthermore, dscNanoATAC enables allele-specific co-accessibility inferences (Fig. 2c), offering additional insights into allele-specific regulatory interactions.

### Deciphering epigenetic landscapes in complex genomic regions

Repetitive DNA sequences account for ~50% of the human genome, and their activity is typically tightly regulated^25,26^. Recent studies have revealed that certain transposable elements (TEs) can also participate in gene regulation under certain conditions^27–29^. However, short-read sequencing-based methods struggle to distinguish individual repetitive elements because of their inability to uniquely map reads to these genomic loci^14^. In contrast, long-read sequencing methods can overcome this limitation by simultaneously capturing individual repetitive elements and their unique flanking regions. Leveraging this capability, we used dscNanoATAC to more accurately detect complex genomic regions. Compared with those identified by short-read sequencing, the proportion of peaks identified by dscNanoATAC that were located in low-mappability regions (150 bp Mappability < 0.5; see Materials and Methods) increased twofold (1% vs 2%) (Supplementary Fig. S8a and Table S4). By calculating the genome-wide mappability for 150 bp and 3 kb reads and estimating the average mappability of each TE copy, we found that long reads significantly outperformed short reads in mapping TEs (Supplementary Fig. S8b). When the length of the TE exceeds 1 kb, the mapping efficiency of short reads rapidly decreases, whereas that of long reads remains largely unaffected (Supplementary Fig. S8c). Therefore, long reads have distinct advantages in mapping longer TEs such as long terminal repeats (LTRs), long interspersed nuclear elements (LINEs), and satellites (Supplementary Fig. S8d). Indeed, when dscNanoATAC was compared with the short-read-based method, although the overall distribution of the identified peaks within TEs did not significantly differ, dscNanoATAC was significantly more efficient in detecting various TE families in low-mappability regions (Supplementary Fig. S8e–g). dscNanoATAC also identified accessible peaks located within TEs that possess potential regulatory functions. For example, we detected an accessible peak at an L2b element (LINE) upstream of the chemokine gene *XCL1*, which exhibited co-accessibility with the *XCL1* promoter, suggesting a potential regulatory relationship between the two elements (Fig. 2f). We also detected accessible peaks within a mammalian-wide interspersed repeat element, a type of short interspersed nuclear element (SINE), upstream of *FCGR2B* and an L1PA5 (LINE) element upstream of *CD80*, all of which were co-accessible with their promoters (Supplementary Fig. S8h). These results demonstrate that dscNanoATAC can accurately capture accessibility signals in complex genomic regions and reveal the potential regulatory roles of the latter.

### Allele-specific-C.Origami enables the prediction of high-resolution cell type- and allele-specific 3D chromatin architectures

**Fig. 3:**
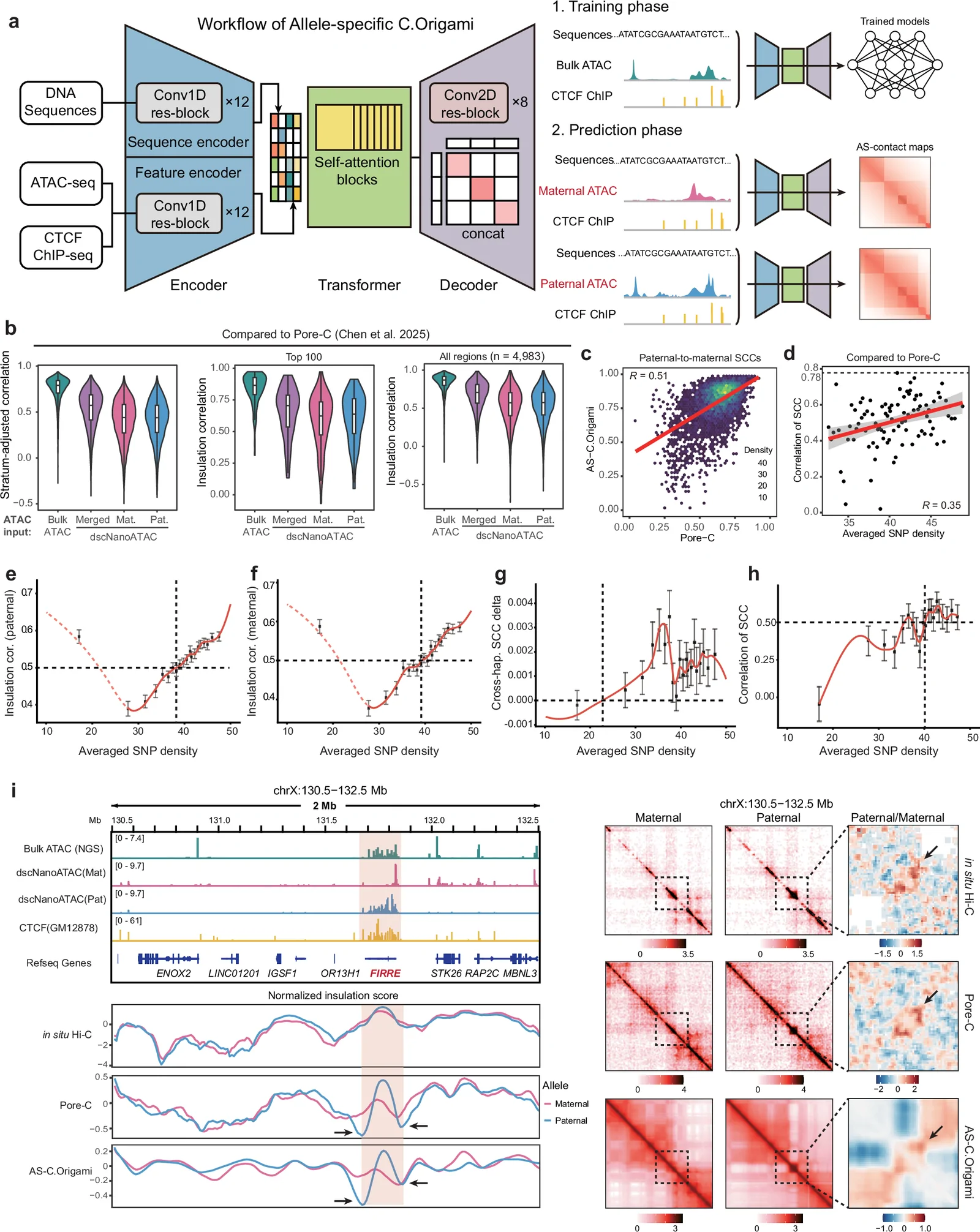
AS-C.Origami enables the cell type- and allele-specific prediction of the 3D chromatin architecture. **a** Schematic of the AS-C.Origami workflow. **b** SCC and correlation coefficients of insulation scores between predicted and experimental results (Pore-C data)^34^. The predictions were based on bulk ATAC-seq, merged, paternal, and maternal dscNanoATAC data. **c**. Correlation analysis of paternal-to-maternal SCCs between AS-C.Origami and Pore-C data (*n* = 4983). **d** Correlation analysis of paternal-to-maternal SCCs between AS-C.Origami and Pore-C data with different SNP densities. A total of 4950 2-Mb genomic regions were sorted by SNP density and divided into groups of 50 to calculate correlation coefficients separately for each group. **e**, **f** Correlation analysis of insulation scores between the predicted and experimental results (Pore-C) for the paternal (**e**) and maternal (**f**) alleles. **g** Differences in the SCC across haplotypes. **h** Pearson’s correlation coefficient of the SCCs between parental haplotypes of the predicted and experimental results. All 2-Mb regions with an SNP density > 0 (*n* = 5780) were used. Regions were binned with the same number (*n* = 289). Note that the unusually high insulation score correlation coefficient in the first bin may be attributed to the fact that most regions in these bins have not available values for insulation scores, and the few remaining regions with extremely low SNP density may not significantly differ between the parental haplotypes. **i** Genome browser view showing allele-specific accessibility (left top), associated insulation profiles (left bottom), and contact maps from bulk Hi-C, Pore-C, and AS-C.Origami (right) at the *FIRRE* locus. In the right panel, the merged, maternal and paternal contact maps and the differential contact map between the paternal and maternal alleles (red and blue indicating paternal- and maternal-specific interactions, respectively) are shown. The differential structures between parental alleles are highlighted with arrows.

The intricate 3D architecture of the genome plays a central role in gene regulation through the organization of regulatory elements into functional networks^30^. Although significant progress has been made in the field of 3D genomics, how allele-specific chromatin structures are formed and what their regulatory functions are remain unclear. Owing to the inherent sparsity of Hi-C data, the detection of cell type- and allele-specific interactions at high resolution remains a significant challenge. Building upon a previous study^31^, we developed Allele-specific-C.Origami (AS-C.Origami), a deep-learning-based workflow that predicts allele-specific 3D chromatin contact maps directly from dscNanoATAC data. Specifically, we first trained a C.Origami model of GM12878 cells on the basis of DNA sequence, bulk ATAC-seq, and CTCF ChIP-seq data, and Hi-C data as training targets. We then predicted the allele-specific contact maps by using dscNanoATAC GM12878 data as inputs (Fig. 3a). Owing to the difficulty in obtaining high-resolution, allele-specific Hi-C data, a gold-standard dataset is currently lacking. Although we used deeply sequenced bulk in situ Hi-C data (~826 Gb of raw data, 4 billion contacts)^32^ to perform haplotype tagging, the generated allele-specific contact maps were still highly sparse and noisy. In contrast, compared with in situ Hi-C, AS-C.Origami generated much denser allele-specific contact maps. To systematically benchmark our results while accounting for the lack of available SNP sites, we first identified 2-Mb genomic windows of sufficient SNP density (*n* = 4983; see Materials and Methods) and calculated the stratum-adjusted correlation coefficients (SCCs)^33^ and correlation coefficients of insulation scores between the experimental and predicted allele-specific contact maps (Supplementary Fig. S9a). We also included Nanopore sequencing-based Pore-C data from a recent study^34^ as an example state-of-the-art for allele-specific contact maps to further mitigate the impact of SNP sparsity. Our predictions were more similar to the Pore-C data than the bulk Hi-C data, as the average SCCs increased from 0.32 to 0.41 (*n* = 4983), whereas the average correlation coefficients of the insulation score increased from ~0.41 to 0.54 (*n* = 4983) (Fig. 3b; Supplementary Fig. S9a). We also showed that the correlation strengths of the insulation scores for allele-specific contact maps were positively correlated with SNP density (Pearson’s *R* = 0.12) and could be further improved to ~0.60 when only the top 100 regions with the highest SNP density were evaluated (Fig. 3b; Supplementary Fig. S9b).

To further demonstrate the ability of AS-C.Origami to capture the structural differences between paternal and maternal alleles, we subsequently calculated the SCCs between the paternal and maternal contact maps and assessed the correlations of the paternal-to-maternal SCCs between the experimental and predicted data to evaluate their consistency in reflecting allele-specific differences. Consequently, we showed a strong correlation between the SCCs of the experimental and predicted allelic interaction variations (Pearson’s *R* = 0.51, *n* = 4983) (Fig. 3c; Supplementary Fig. S9c). The correlation coefficient further increased to 0.78 when regions with higher SNP density were considered (Fig. 3d; Supplementary Fig. S9d). By assessing how these metrics changed with SNP density, we considered the predictions reliable when at least 80% of the 10-kb bins in a 2-Mb window contained one or more SNP sites, as the correlation coefficients of both the insulation and the SCC exceeded 0.5 (Fig. 3e–h). In GM12878, such regions accounted for 47% of the genome (2820 out of 6004 2-Mb sliding windows). We also demonstrated that the inclusion of CTCF data was important for increasing the prediction performance, whereas the use of heterozygous SNP sites for generating haplotype-specific DNA sequences or CTCF signals did not further improve model performance (Supplementary Fig. S10). These results indicated that non-SNP factors, such as epigenetic imprinting, likely exert a relatively strong influence on haplotype-specific chromatin structures.

Subsequently, we identified the allele-specific topologically associating domain (TAD) boundaries using Pore-C data and tested the performance of our model in these regions. Consistent with the paternal X chromosome inactivation reported in the GM12878 cell line, we revealed significant enrichment of allele-specific TADs within the X chromosome. For example, we identified a strong paternal-specific domain in the *FIRRE* locus, which is known to form a super-loop with the *DXZ4* locus and is important for maintaining X chromosome inactivation^35,36^. Consistently, this paternal-specific domain was successfully captured by our model, as indicated by the differential contact maps and insulation profiles generated from our data (Fig. 3i). Similarly, we showed that multiple allele-specific boundaries in the X chromosome could also be captured by our method. However, these patterns were less prominent in the bulk Hi-C data, as clear insulation valleys could only be observed in the results from Pore-C and AS-C.Origami results indicating that compared with the original in situ Hi-C, AS-C.Origami predicted better allele-specific 3D structures in these regions (Fig. 3i; Supplementary Fig. S11a, b). We also applied the AS-C.Origami workflow to known imprinted regions in the GM12878 cell line. We showed that AS-C.Origami successfully captured paternal-specific interactions between the *SNRPN/SNURF* and *UBE3A* genes, which are located in a known maternally imprinted region and are specifically expressed in the paternal allele (Supplementary Fig. S11c). Together, these results highlight the ability of AS-C.Origami to predict allele-specific regulations, suggesting that the integration of a deep-learning framework with allele-specific chromatin accessibility data can advance our understanding of the allele-specific 3D genomic organization.

### dscNanoATAC revealed allele-specific regulatory elements in the mouse brain

**Fig. 4:**
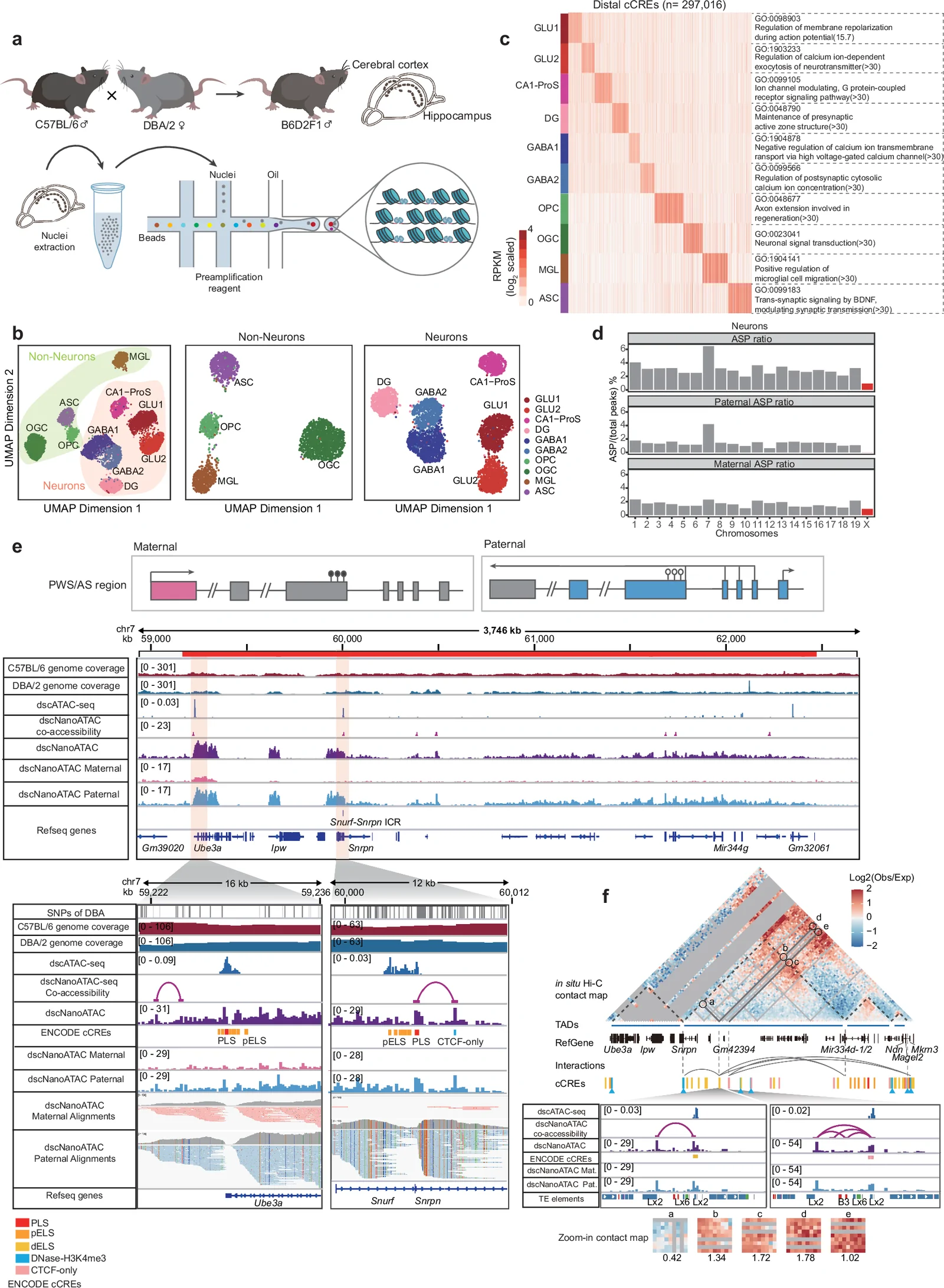
dscNanoATAC identified allele-specific regulation in the mouse brain. **a** Workflow of dscNanoATAC in adult mouse CTX and HPF samples. **b** UMAP showing the clustering of single nuclei from adult mouse CTX and HPF samples. From left to right: all clusters, non-neuronal clusters and neuron clusters. **c** Distal cCRE modules of each cell type in the mouse brain. Representative GREAT enrichment results are indicated on the right, and values in parentheses indicate the −log_10_(*P* value) of the related GO term. **d** Distribution of the proportion of ASPs in neurons of the mouse brain across each chromosome. **e** Genome browser view of the Prader–Willi syndrome and Angelman syndrome (PWS/AS) region, indicating the allele-specific regulation of *Ube3a* in neurons. From top to bottom: genome coverage for C57BL/6 (maternal) and DBA/2 (paternal) samples; 10x ATAC-seq data of neurons; co-accessibility identified by dscNanoATAC; dscNanoATAC data of the merged, maternal and paternal signals in the neurons; and RefSeq gene annotations. The *Ube3a* and *Snurf* loci are highlighted in the box with the haplotype-resolved alignments of dscNanoATAC. **f** Two pairs of Lx2-associated peaks were detected in the PWS/AS regions, indicating interactions between *Snrpn* and downstream imprinting genes. From top to bottom: observed-to-expected mouse cerebral in situ Hi-C map; TADs; RefSeq gene annotations; interactions estimated from Hi-C data; ENCODE cCREs; and magnified regions of the Lx2-associated peaks. The five interaction loci indicated in the top Hi-C map are magnified at the bottom.

To demonstrate the ability of dscNanoATAC to analyze heterogeneous and complex tissues, we applied dscNanoATAC to fresh-frozen cerebral cortex (CTX) and hippocampal formation (HPF) samples from adult mice, with one independent experimental reaction per tissue (Fig. 4a). After strict filtering, a total of 7621 cells were successfully captured, including 3125 cells from the CTX samples and 4496 cells from the HPF samples. Even at a shallow sequencing depth (~0.01 Gb/cell and a median of ~3000 reads), we obtained an average of 2182 unique fragments per cell (with a median duplication rate of 27%) (Supplementary Fig. S12a, b and Table S1). By performing unsupervised clustering and label transfer using reference datasets^12,37^, we showed that all major cell types in the CTX and HPF samples could be captured by dscNanoATAC, including layer 2/3/4 intra-telencephalic (L2/3/4 IT, GLU1, *Cux2*) and layer 5 pyramidal subclasses (L5 PT, GLU2, *Fezf2*) of glutamatergic excitatory neurons (GLU1-2, *Neurod6*); Sst (GABA1, *Sst*) and Pvalb (GABA2, *Pvalb*) subclasses of GABAergic inhibitory neurons (GABA1-2, *Gad2*); oligodendrocytes (OGC, *Mobp*); microglia (MGL, *C1qb*); oligodendrocyte progenitor cells (OPC, *Pdgfra*); and astrocytes (ASC, *Pax6*) (Fig. 4b; Supplementary Fig. S12c–g). We also identified two HPF-specific neuron subtypes, CA1-Prosubiculum neurons (CA1-ProS, *Lefty1*) and dentate gyrus neurons (DG, *Prox1*), consistent with previous results^12^ (Supplementary Fig. S12c). To evaluate the ability of dscNanoATAC to resolve cCRE activity, we subsequently examined accessibility variations using previously identified cCREs from different cell types^38^. By performing non-negative matrix factorization (NMF), we identified 10 cCRE modules for proximal and distal cCREs separately, revealing their cell-type specificity (Fig. 4c; Supplementary Fig. S12h and Table S5). The results of Gene Ontology (GO) analysis further confirmed the functional relevance of these cCRE modules. For example, the cCRE modules of GLU were enriched in regulation of synaptic vesicle transport, whereas the cCRE module of MGL was enriched in regulation of antigen processing and presentation of peptide antigen (Fig. 4c; Supplementary Fig. S12h).

We subsequently performed genome-wide analyses of allele-specific peaks and neighboring co-accessibility for neural and non-neural cells, respectively. Among the 124,959 and 60,320 peaks identified for neural and non-neural cells, 3893 (1820 paternal; 2073 maternal) and 835 (399 paternal; 436 maternal) peaks exhibited allele-specific accessibility, accounting for 3.1% and 1.4% of the total peaks, respectively (Fig. 4d; Supplementary Fig. S13a, b and Table S6). We also detected 4683 pairs of peaks that demonstrated significant co-accessibility patterns in neurons, including those corresponding to the neuron-specific genes *Slc17a6*, *Kcnv1*, and *Camk4* (Supplementary Fig. S13c, d and Table S7). In addition to the canonical allele-specific imprinting genes such as *Zdbf2* and *Meg3* (Supplementary Fig. S13e), we identified the significant enrichment of paternal-specific peaks in neuronal chr7, with the majority localized to chr7C, an imprinting region homologous to the human 15q11–q13 locus, which is associated with two opposing neurodevelopmental disorders and is known as the Prader–Willi syndrome and Angelman syndrome (PWS/AS) region^39–41^ (Fig. 4d). Within this region, *Ndn*, *Snrpn-Snurf*, *Ipw*, and several small nucleolar RNAs (snoRNAs) were paternally expressed. In contrast, *Ube3a* was maternally expressed in neurons and was regulated by an antisense RNA (*Ube3a-as*) as part of the long non-coding transcript *Snhg14*, which originates from the promoter of *Snrpn*^39,40^. During early brain development, *Ube3a* is initially biallelically expressed in neurons. As the neurons mature, the paternal *Ube3a-as* is upregulated, leading to silencing of the paternal *Ube3a* allele and the establishment of maternal expression^41^. Our results consistently demonstrated that the entire PWS/AS region exhibited allele-specific chromatin accessibility, with the paternal allele showing broad accessibility across the locus. In contrast, only the *Ube3a* locus was accessible in the maternal allele (Fig. 4e). Notably, the genomic region corresponding to the *Snhg14* locus exhibited significantly increased accessibility in the paternal allele. Using the long-read whole-genome sequencing data^42^, we further ruled out the possibility that this phenomenon is caused by strain-specific copy number variations, instead considering that it likely reflects active transcription of the paternal *Snhg14/Ube3a-as* (Fig. 4e). We also identified multiple neighboring co-accessibility events within this region, including a significant interaction between a predicted CTCF-binding site (annotated by ENCODE cCREs) and the *Snurf* promoter (which also serves as the major promoter of *Snhg14*) in the paternal allele (Fig. 4e; Supplementary Fig. S13f), which suggests the potential role of chromatin structure in the allele-specific regulation of *Snurf* imprinting. We confirmed that these allele-specific accessibility patterns within the PWS/AS region were specifically observed in all subtypes of neurons from both the CTX and HPF samples, while both the paternal and maternal alleles of non-neuronal cell types were not accessible (Supplementary Fig. S14). In summary, dscNanoATAC shows great potential in its ability to perform high-resolution allele-specific chromatin accessibility analysis in complex tissues and to identify allele-specific regulatory mechanisms.

Additionally, we leveraged the ability of dscNanoATAC to detect complex genomic regions to identify transposon-associated regulatory elements in our mouse brain dataset. In agreement with prior observations in the human genome, we found that long reads (3 kb) significantly outperformed short reads (150 bp) in identifying relatively long repetitive elements, such as LINEs and LTRs, within the mouse genome (Supplementary Fig. S15a–c). Notably, the proportion of peaks detected in low-mappability regions in our dataset was approximately double that of the short-read-based methods, and enrichment of these peaks within LINEs, LTRs, and SINEs was markedly greater than that with the other methods (Supplementary Fig. S15d–g and Table S8). Interestingly, we identified multiple genes linked to neural function that may be regulated by transposable elements. For example, we identified an accessible chromatin peak within a B1F element upstream of the *Nptn* gene, which exhibited co-accessibility with the *Nptn* promoter, indicating a potential regulatory interaction between the two (Supplementary Fig. S15h). The *Nptn* gene has been implicated in the assembly of hippocampal excitatory synapses. Interestingly, we also identified two paternal-specific peaks within the PWS/AS region located at Lx2 elements (belonging to the LINE-1 family) that exhibited neighboring co-accessibility; these features were only partially detectable using short-read-based methods (Fig. 4f). These two peaks also separately overlapped with predicted distal enhancer-like signature (dELS) and DNase-H3K4me3 elements in ENCODE cCREs, suggesting their potential regulatory activities. To further demonstrate their potential functions, we included previously published mouse cerebral Hi-C datasets to reveal the interactions of these elements with other sections in the PWS/AS region^43^. Unexpectedly, we showed that in addition to the interaction networks between known CTCF sites, these two pairs of peaks were enriched in interactions with downstream TAD boundaries near *Mir334d-1/2* and *Magel2*. One of the peak pairs also showed a weak interaction with the *Snrpn* imprinting region (Fig. 4f). Previous studies have shown that the *Snrpn* imprinting center can form allele-specific interactions with downstream genes, including *Magel2* and *Mkrn3*, for expression control^44,45^. Our results indicated that these two pairs of Lx2-associated peaks may also be involved in this allele-specific regulatory function. Taken together, these findings emphasize the ability of dscNanoATAC to reveal regulatory elements that were previously undetectable with short-read-based methods, thereby elucidating the potential regulatory roles of these elements in cell type-specific activities.

### dscNanoCUT: a high-throughput tool for detecting allele-specific histone modifications

**Fig. 5:**
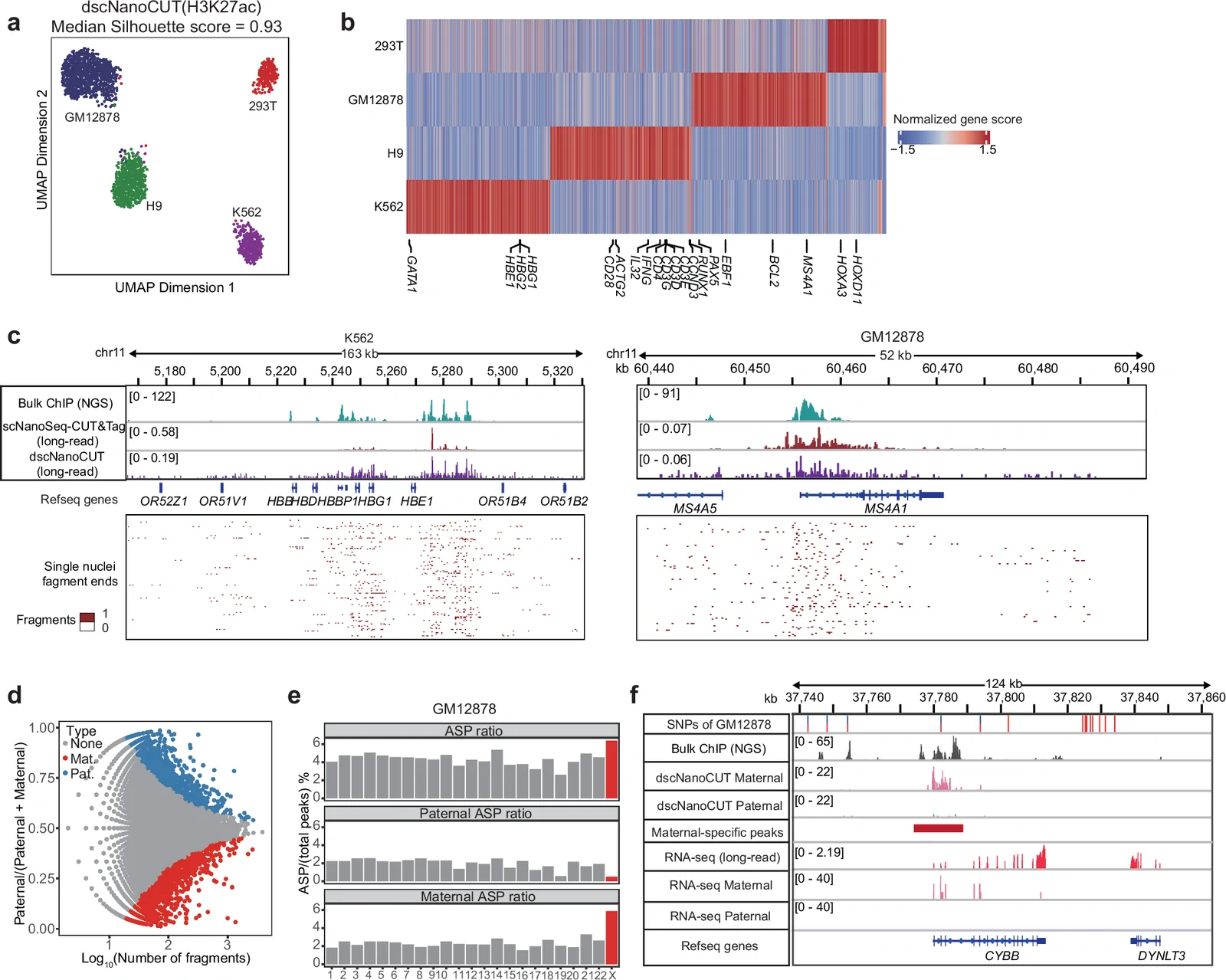
dscNanoCUT captured allele-specific histone modifications. **a** UMAP of four cell lines according to dscNanoCUT. The silhouette coefficient is calculated on the basis of the top 2 to 5 LSI dimensions. **b** Heatmaps showing the gene scores of cell type-specific markers in the four human cell lines. **c** Genome browser view showing representative regions of pseudo-bulk and single-cell H3K27ac histone modification signals from dscNanoCUT in K562 and GM12878 cells, along with bulk ChIP-seq and scNanoseq-CUT&Tag data^17^. The associated genes, *HBG1* and *MS4A1*, are marker genes for K562 and GM12878, respectively. **d** Ratio between paternal fragments and the total number of haplotyped reads for each peak in GM12878 identified by H3K27ac dscNanoCUT. Statistically significant ASPs (FDR < 0.05) are highlighted in blue (paternal) and red (maternal). **e** Distribution of H3K27ac ASPs in GM12878 across chromosomes. **f** Genome browser view showing examples of identified and previously known X chromosome inactive genes, such as *CYBB*, with maternal-specific H3K27ac peaks and expression.

After validating the feasibility of dscNanoATAC, we extended our approach to profile H3K27ac modifications by applying dscNanoCUT to a mixture of four cultured human cell lines. Following quality filtering, 2100 high-quality single cells were retained, comprising 928 GM12878, 602 H9, 343 K562, and 227 HEK293T cells. The dscNanoCUT data demonstrated high reproducibility between replicates (Pearson’s *R* = 0.70) (Supplementary Fig. S16a). Using UMAP dimensionality reduction, the four cell lines were distinctly clustered (median silhouette score = 0.93) and could be accurately distinguished on the basis of the gene scores of canonical marker genes (Fig. 5a–c; Supplementary Fig. S16b–d). We also systematically benchmarked dscNanoCUT against other single-cell CUT&Tag technologies^8,9,17^. At a relatively low sequencing depth ( ~0.01 Gb/cell), dscNanoCUT generated an average of 3346 unique fragments per cell, comparable to that of droplet-based CUT&Tag methods employing short-read sequencing platforms, while maintaining a moderate duplication rate. Notably, the FRiP of dscNanoCUT exceeded that of scNanoSeq-CUT&Tag, indicating a greater signal-to-noise ratio (Supplementary Fig. S17a–c). To further evaluate the peak-calling performance of our method, we compared the peaks identified by scNanoSeq-CUT&Tag and dscNanoCUT using ENCODE ChIP-seq peaks as a reference. The outputs of both methods showed comparable overlaps with the ENCODE peaks (Supplementary Fig. S17d). Analysis of the distribution of the peaks within the ENCODE cCREs and the RefGene annotations revealed that, compared with scNanoSeq-CUT&Tag, dscNanoCUT was slightly more enriched in cCREs (Supplementary Fig. S17e, f). These findings verified the ability of dscNanoCUT to profile single-cell chromatin modifications.

Like the previously described dscNanoATAC, dscNanoCUT can efficiently capture heterozygous SNPs. Leveraging dscNanoCUT data from the GM12878 cells, we identified ASPs and found that 5839 out of the 49,071 total peaks exhibited allele specificity (Fig. 5d; Supplementary Table S9). Consistent with previous results, compared with those on the autosomes, the frequency of ASPs on the X chromosome was significantly greater, with a predominant bias toward maternal alleles (Fig. 5e). Specifically, we identified 44 known X chromosome inactivation genes that exhibited maternal-specific H3K27ac peaks and corresponding gene expression patterns^22^ (Supplementary Fig. S17g and Table S9). A representative example is the *CYBB* gene, where H3K27ac peaks in its promoter region were exclusively detected in the maternal allele, which is consistent with its maternal-specific gene expression profile (Fig. 5f). These findings validate the effectiveness of dscNanoCUT in highly precisely detecting allele-specific protein-binding signals.

**Fig. 6:**
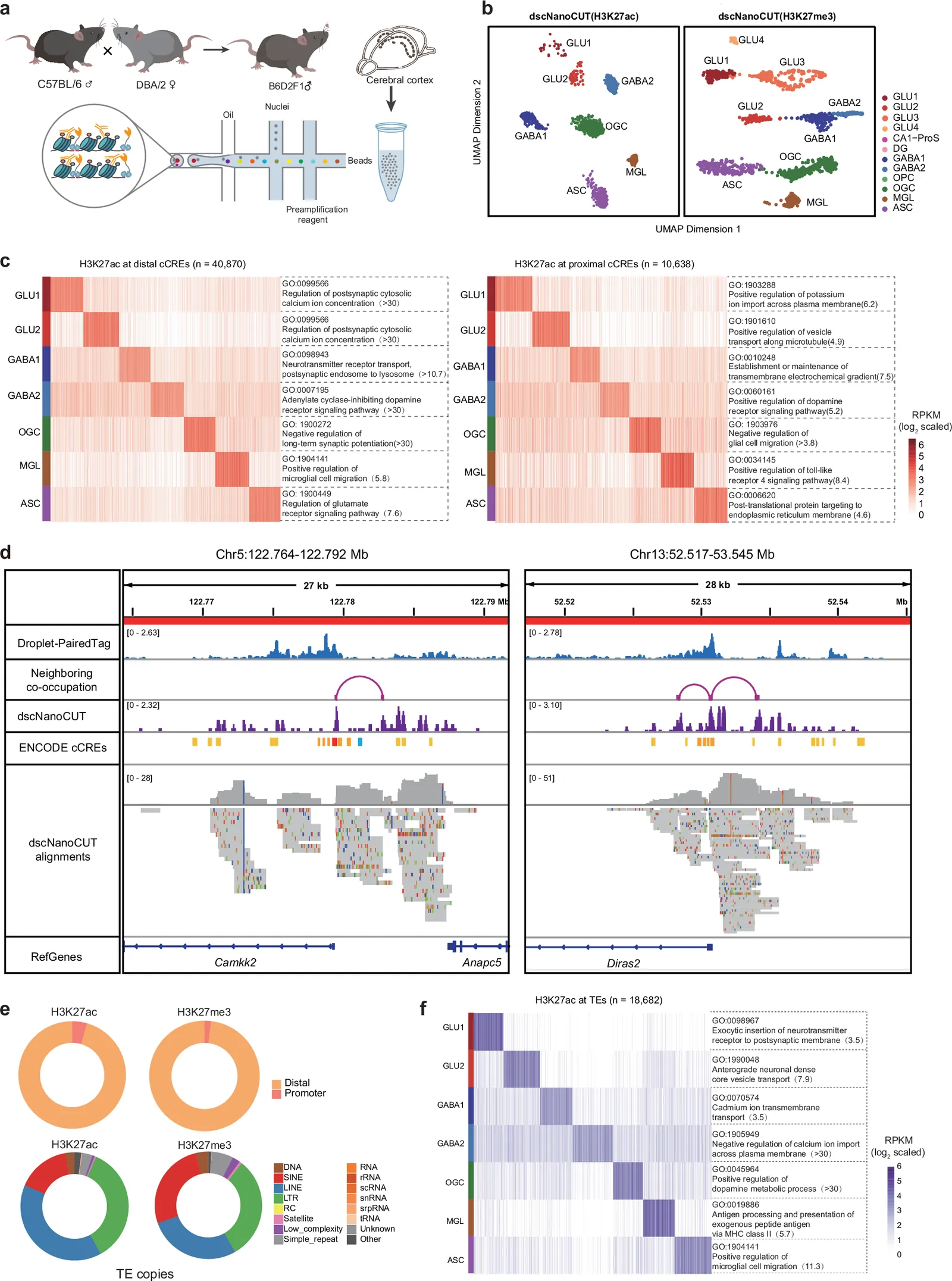
dscNanoCUT captured allele-specific histone modifications in the mouse brain. **a** Workflow of dscNanoCUT with adult mouse CTX samples. **b** UMAP showing the clustering of single nuclei from adult mouse CTX samples. H3K27ac modifications are shown on the left; H3K27me3 modification are shown on the right. **c** Distal (left) and proximal (right) cCRE modules of each cell type in the mouse brain according to H3K27ac modification. Representative GREAT enrichment results are indicated on the right, and values in parentheses indicate the −log_10_(*P* value) of the related GO term. **d** Genome browser view of representative H3K27ac co-occupation events at the *Camkk2* and *Diras2* loci. **e** Proportion of detected TEs located in distal or proximal regions (above) and the percentage of TE origins, according to H3K27ac and H3K27me3 modifications. **f** H3K27ac-marked TE modules of each cell type in the mouse brain. Representative GREAT enrichment results are indicated on the right, and values in parentheses indicate the −log_10_(*P* value) of the related GO term.

To demonstrate the ability of dscNanoCUT to analyze the single-cell epigenome of complex primary tissues, we then profiled both H3K27ac and H3K27me3 modifications in the mouse CTX with our proposed method. After filtration, 1946 and 1884 cells corresponding to the H3K27ac and H3K27me3 modifications, respectively, were recovered (Supplementary Fig. S18a, b). We then performed integrative analyses with published datasets and annotated cell types of identified clusters on the basis of their paired single-cell RNA-seq data^12^. We showed that both the H3K27ac and H3K27me3 modifications identified by dscNanoCUT successfully captured all the major cell types in the mouse cortex (Fig. 6a, b), and the identified cell clusters in our datasets were strongly correlated with previous results in terms of both gene scores and genome-wide signals (Supplementary Fig. S18c–g). We also showed that dscNanoCUT could identify allele-specific modifications in both neurons and non-neuron cells (Supplementary Fig. S19a, b). To further demonstrate the ability of dscNanoCUT to characterize the epigenetic states of cCREs, we analyzed variations in previously identified cCREs among different cell types^38^ and classified them into modules on the basis of their cell-type specificity (Fig. 6c; Supplementary Fig. S19c). To this end, 51,508 (10,638 proximal; 40,870 distal) and 2909 (510 proximal and 2399 distal) cCREs with sufficient H3K27ac and H3K27me3 levels were identified. Consistent with previous results, dscNanoCUT identified cell type-specific cCRE modules and reveal their relationships to related pathways. For example, H3K27ac distal cCRE modules of microglia were enriched in positive regulation of microglial cell migration, whereas those of glutamatergic neurons were enriched in regulation of postsynaptic cytosolic calcium ion concentration (Fig. 6c; Supplementary Fig. S19c and Table S10). The results of co-occupancy analyses also showed that some of these events reflected interactions between promoters and enhancers. For example, we identified H3K27ac co-occupation between the promoter and upstream enhancers of *Camkk2* in neurons, which encodes a Ca^2+^/calmodulin (CaM)-dependent protein kinase that is highly expressed in brain neurons and plays critical roles in regulating energy metabolism and neuronal processes. We also identified several gene–enhancer pairs in genes that are highly expressed in neurons and play important roles in neuronal development and functions, such as *Diras2*, *Plxna2*, and *Rab6b* (Fig. 6d; Supplementary Fig. S19c, d). These results indicated that dscNanoCUT may facilitate the identification of gene–enhancer pairs according to co-occupancy.

Given the advantages of dscNanoCUT in analyzing low-mappability regions, we next focused on TE activity. We first quantified the number of histone modifications on each TE copy in each cell. To avoid confounding gene signals, all the TE copies that overlapped with gene bodies were excluded from downstream analyses. When we performed unsupervised clustering, we observed that both the H3K27ac and H3K27me3 modifications of TEs could be used to roughly divide cells into neuronal and non-neuronal groups, suggesting that the two groups have different TE epigenetic profiles (Supplementary Fig. S19e). We then selected TE copies with sufficient H3K27ac or H3K27me3 signals (reads per kilobase per million mapped reads (RPKM) > 1 in at least one cell type and detected in > 3 cells) to detect NMF modules. We showed that most of these TEs were located in the distal region of genes and had different origins depending on their modifications; the TEs with H3K27ac modifications were mainly from the LINE and LTR classes, whereas those with H3K27me3 modifications were mainly from the SINE, LINE and LTR classes (Fig. 6e). Moreover, by classifying these TEs into modules, we demonstrated the cell type specificity of their epigenetic statuses (Fig. 6f; Supplementary Fig. S19f). Through GREAT enrichment analysis, we found that cell-specific H3K27ac-modified TE modules were often associated with cell type-specific functions. For example, the MGL-specific TE module was enriched in antigen processing and presentation of exogenous peptide antigen via MHC class II, whereas the TE module of GABA1 was enriched in cadmium ion transmembrane transport. In contrast, H3K27me3-modified TE modules were generally enriched in development or immune pathways (Fig. 6f; Supplementary Fig. S19f and Table S11). These findings align with those of previous studies suggesting that certain TEs may serve as enhancers for activating the expression of cell type-specific genes^46–48^. Taken together, our results demonstrate the ability of dscNanoCUT to elucidate the epigenetic statuses of TEs in complex primary tissues. Future studies are warranted to investigate the underlying mechanisms involved.

## Discussion

In this study, we present dscNanoATAC and dscNanoCUT, the first droplet-based microfluidics techniques adapted to Nanopore long-read epigenome sequencing, which enable scalable and cost-effective single-cell epigenomic profiling. This strategy could also be easily applied to other droplet-based microfluidic platforms by redesigning the Tn5 adapter and amplification primer according to bead oligos (see examples in Supplementary Fig. S20).

A key strength of our approach is its ability to characterize cell type-specific allelic regulatory mechanisms within complex tissues. By leveraging the inherent advantages of long-read sequencing, including high haplotype tagging efficiency and the ability to resolve repetitive elements, we successfully mapped allele-specific chromatin accessibility and histone modifications across diverse cell populations. Moreover, through the identification of neighboring co-accessibility events, our methods allow the estimation of potential regulatory relationships between cCREs and their nearby genes, which could be important for understanding the functional impact of non-coding variants. Compared with previous correlation-based co-accessibility methods, this approach emphasizes the most important relationships, thereby reducing potential false positives in co-accessibility estimation. By integrating neighboring co-accessibility events with multiway interactions identified from scNanoHi-C data, we constructed a hierarchical network of enhancer–promoter interactions, which may serve as the structural basis for enhancer cooperativity to confer their synergistic effects on gene regulation events.

By applying dscNanoATAC and dscNanoCUT to mouse brain samples, we demonstrated the ability of our methods to contend with the heterogeneity of complex tissues, showing that both dscNanoATAC and dscNanoCUT were able to identify cell type-specific cCRE modules. Notably, we precisely delineated the allele-specific regulation of *Ube3a* within the imprinted PWS/AS region in the neurons of the mouse brain. We identified interactions between a CTCF-like cCRE and the *Snurf* promoter and revealed two pairs of Lx2-associated peaks that interact with *Snurf* and downstream imprinting genes. These observations are consistent with the results of recent work in human neurons, which revealed that the CTCF anchor near *SNURF* is paternally hypomethylated to enable loop formation with its upstream regions^45^.

Recent studies have reported that different classes of TEs function as enhancers to activate the expression of nearby genes and affect cell fate during multiple biological processes^47,48^. With dscNanoCUT, we further evaluated the activity of TEs in the mouse brain and revealed that the H3K27ac-modified TE modules were related to cell type-specific pathways, highlighting the potential roles of these TEs as regulatory elements. Future studies are warranted to further validate the functions of these elements.

Determining allele-specific 3D chromatin structures is crucial for understanding the function of CREs. However, current chromatin conformation capture methods still have significant limitations in this regard. First, Hi-C technology relies on the detection of chimeric fragments, which greatly restricts the sequencing length of individual fragments that can be analyzed. Even with our previously developed scNanoHi-C method^18^, although the read length can reach ~3 kb, the length of individual fragments is only ~700 bp, resulting in a limited capture rate for the SNP sites necessary for distinguishing parental alleles. Moreover, the inherent sparsity of heterozygous SNP sites further limits the resolution of allele-specific Hi-C, especially for human-derived samples. These challenges underscore the limited ability of current Hi-C technologies to detect allele-specific chromatin structures. By leveraging the high haplotype tagging percentage of the dscNanoATAC data (~60%), we developed the AS-C.Origami workflow on the basis of a previous deep learning model to predict high-resolution, allele-specific chromatin architectures, overcoming the sparsity-based limitations of conventional Hi-C and achieving a 10-kb resolution in human cell line data. This strategy provides a conceptual advancement that may inspire further studies in the development of deep-learning-based methods for revealing the allele-specific effects of 3D structures, which would be especially useful for understanding the mechanisms of gene imprinting and the functions of CREs. Recently, a similar work reported Fiberfold, a model that was trained exclusively on Fiber-seq data that enables the external-data-free prediction of haplotype-specific chromatin structures^49^. In comparison, AS-C.Origami, which leverages single-cell data, is better suited for contending with cellular heterogeneity. Our methods establish a comprehensive epigenomic landscape spanning chromatin accessibility, histone modification, and higher-order chromatin structures while providing insights into the interplay between chromatin states and 3D genome structures.

In conclusion, our work establishes a droplet-based high-throughput framework for single-cell epigenomics using a long-read sequencing platform, providing insights into cellular heterogeneity, allelic regulation, and their interplay with 3D genome organization within complex tissues. With the development of long-read sequencing and continued reductions in sequencing costs, we believe that our methods hold immense potential for widespread use and may increase our understanding of the intricate regulatory network underlying the development of disease processes.

### Limitations of the study

Our study has several limitations. First, the read length and throughput of our methods could be further improved. In this respect, optimizing the Tn5 enzyme concentration and microfluidic pre-amplification systems may increase the amplification efficiency and read length. Additionally, the throughput could be further increased by incorporating multi-barcoded Tn5 prior to microfluidic processing. Second, our methods currently capture only epigenomic data and lack matched gene expression information, preventing direct assessments of the effects of chromatin accessibility and histone modification on transcription. However, given their compatibility with commercial microfluidic systems, our methods could be integrated with other multi-omic platforms to resolve this issue in the future. Third, owing to the relatively long reads, our method yields fewer fragments than short-read-based approaches do at the same sequencing depth. Nevertheless, the extended read length enables us to efficiently detect allele-specific regulation and complex genomic regions. We believe that as sequencing costs continue to decrease, our methods will hold increasing potential for broader application. Fourth, the cell recovery rate of dscNanoCUT could be improved, as the lack of a dedicated reverse cross-linking step in the current microfluidic system may limit amplification efficiency. Finally, the AS-C.Origami workflow currently uses only dscNanoATAC data. Integrating dscNanoCUT data into a unified deep learning framework may further improve allele-specific structure predictions. Future work could also employ allele-specific in silico perturbations to identify genetic and epigenetic contributions to regulatory variability and enable predictive modeling across different cellular contexts.

## Materials and methods

### Cell culture

GM12878 (HG001, purchased from the Coriell Institute) was cultured in RPMI-1640 (Gibco, Cat# 11875093) supplemented with 15% fetal bovine serum (FBS; Gibco, Cat# 26140079) and 1% penicillin/streptomycin (PS; Gibco, Cat# 15140122). K562 cells (purchased from the National Infrastructure of Cell Line Resource) were cultured in RPMI-1640 supplemented with 10% FBS, 1% GlutaMAX (Gibco, Cat# 35050061), and 1% PS. H9 cells (purchased from the National Infrastructure of Cell Line Resource) were cultured in RPMI 1640 supplemented with 10% FBS and 1% PS. 293T cells (purchased from the National Infrastructure of Cell Line Resource) were cultured in DMEM (Gibco, Cat# 11995040) supplemented with 10% FBS and 1% PS. All cell lines were cultured at 37 °C with 5% CO_2_.

### Processing of dissected mouse brain tissues and nuclear extraction

Eight-week-old male B6D2F1/Crl mice were purchased from Charles River Laboratories. The CTX and HPF were dissected in ice-cold PBS, snap-frozen in liquid nitrogen, and stored at −80 °C (or in liquid nitrogen) until the nuclei were isolated. The nuclear extraction protocol was based on that of Tan et al.^50^. First, frozen tissues were placed in 2 mL of nuclear isolation buffer (0.1% Triton X-100, 0.25 M sucrose, 25 mM KCl, 5 mM MgCl_2_, 10 mM Tris, pH 8.0, 1 μM DTT) and homogenized in a 2 mL Dounce homogenizer using 15 strokes with a loose (A) pestle followed by 20 strokes with a tight (B) pestle. The homogenate was centrifuged at 300× *g* for 8 min at 4 °C, and the resulting pellet was resuspended in 1 mL of nuclear storage buffer (0.1665 M sucrose, 5 mM MgCl_2_, 10 mM Tris, pH 8.0) and filtered through a 40 μm strainer. The nuclei were stained with DAPI and sorted using FACS to remove impurities. After sorting, the nuclei were counted and processed using dscNanoATAC.

### dscNanoATAC

First, 100,000–200,000 fresh or frozen cells were centrifuged at 500× *g* for 5 min at 4 °C. Afterward, the pelleted cells were resuspended in Omni-ATAC lysis buffer containing 0.1% IGEPAL CA630 (Sigma, Cat# 18896), 0.1% Tween-20, 0.01% digitonin (Thermo Fisher Scientific, Cat# BN2006), 10 mM Tris-HCl (pH 7.4), 10 mM NaCl, and 3 mM MgCl_2_ and incubated for 3 min on ice. The lysis buffer was diluted with 1× PBS buffer, and the cell nuclei were collected via centrifugation at 800× *g* for 5 min at 4 °C. The nuclei were resuspended in 42 μL of tagmentation buffer (33 mM Tris-acetate (pH 7.8), 66 mM potassium acetate, 10 mM magnesium acetate, 16% dimethylformamide, 0.01% digitonin), 8 μL of 8.3 μM Tn5 transposase mix (Novoprotein, Cat# M045-01B, Novoprotein; the Tn5 adaptor primers are shown in Supplementary Table S12; all primers were synthesized by Sangon Biotech) was added, and then the solution was incubated on a thermomixer at 37 °C and 600 rpm for 30 min. The reaction was stopped with 50 μL of tagmentation stop buffer containing 10 mM Tris-HCl (pH 8.0) and 20 mM EDTA. The nuclei of the tagmented cells were washed with 200 μL of 0.1% BSA-PBS buffer, and the cell nuclei were finally collected using 20 μL of cell suspension buffer (MobiDrop, Cat# S190100101). For further details on the protocol, please refer to the High-throughput single-cell epigenomics technology MobiChIP™ Kit User Guide v1.0 (https://www.mobidrop.com/support/single-cell-reagent/chip-seq).

#### Pre‑amplification master mix preparation

The pre‑amplification master mix was prepared on ice on the basis of the number of chip channels used. The reaction mixture contained 1× pre‑amplification buffer (20.5 μL), pre‑amplification enhancer (4.1 μL), pre‑amplification enzyme (7.7 μL), droplet stabilizers T1 (3.3 μL) and T2 (0.8 μL), cell stabilizer (0.8 μL), and nuclease‑free water (H₂O, 46.8 μL) in a total volume of 84 μL per channel (1.05× excess). After preparation, the mixture was inverted 15 times, centrifuged briefly, and immediately applied to the chip to preserve enzymatic activity.

#### Cell phase reagent preparation

The cell suspensions were adjusted to a concentration corresponding to ~10,000–40,000 cells per loading volume. For loading 45 μL of cell phase reagent, a total of 50 μL was prepared containing (42−N) μL of cell (nucleus) suspension, 0.5 μL of cell stabilizer, 7.5 μL of cell (nucleus) suspension additive, and N μL of cell suspension (where N represents the volume of cell suspension containing the desired cell number). The final cell phase reagent was gently pipetted‑mixed six times with a 45-μL pipette and mixed twice more immediately before loading onto the chip.

#### Chip loading and droplet generation

The chip was seated in the dedicated chip holder, and unused channels (B–E) were blocked by adding 80 μL of 50% glycerol to the corresponding wells. For the active channels, 55 μL of chip beads (equilibrated to room temperature and vortex‑mixed for 10 s), 45 μL of cell-phase reagent, 80 μL of pre‑amplification master mix, and 170 μL of droplet generation oil were sequentially loaded into wells B, C, D, and E, respectively. All the reagents were added slowly to avoid bubble formation. A silicone gasket was then placed over the wells to seal the chip.

#### Droplet harvesting and pre‑processing

After droplet generation, the chip holder was opened, and the clear bottom oil from well A was carefully aspirated using a 200 μL wide‑bore tip at a 70 μL setting while avoiding touching the bottom of the well. The upper white emulsion containing the droplets was then slowly aspirated and transferred along the wall of a 1.5 mL low‑adsorption centrifuge tube. The tube was placed in a droplet preprocessor and run for ~5 min with the blue indicator flashing; the connection between the microspheres and barcode oligos was disrupted using ultraviolet light.

#### Droplet pre‑amplification

After as much bottom oil was removed as possible, 70 μL of the harvested emulsion was transferred into two 0.2 mL PCR tubes each. An 80 μL overlay of the droplet protective agent was gently added on top of the droplets in each tube. The solutions were pre-amplified on a thermal cycler with the following program: 72 °C for 10 min; 98 °C for 3 min; 16 cycles of 98 °C for 20 s, 55 °C for 1 min, and 72 °C for 6 min; a final extension at 72 °C for 5 min; and holding at 4 °C.

#### Post‑amplification processing and emulsion breaking

Following pre‑amplification, 5× resuspension buffer was diluted to 1× with H₂O. The PCR tubes were thawed at 4 °C if previously frozen and centrifuged briefly, after which the protective agent overlay was removed. Each tube was filled with 100 μL of recovery agent and 50 μL of 1× resuspension buffer, mixed by inversion, and centrifuged. The upper emulsion from both tubes was pooled onto a single filter cartridge, followed by an additional washing with 50 μL of 1× resuspension buffer.

The filter cartridge was centrifuged at 10,000× *g* for 5 min. If residual liquid remained, centrifugation was repeated after the cartridge was rotated. The filtrate was retained, and the filter membrane was discarded.

#### Purification of pre‑amplified products and library construction

Pre‑amplified products were cleaned using 1× Ampure XP beads. Finally, the sequencing library was constructed using PCR primers with 24 bp Nanopore barcodes (Supplementary Table S12). We added 34 μL of GXL PCR mix (Takara, Cat# R050A; 5× GXL buffer, 1.5 μL of 10 mM dNTP, 1.5 μL of GXL polymerase, 12 μL of H_2_O, and 4 μL of 10 mM PCR primer) to 16 μL of the product. The following PCR program was then run: 68 °C for 10 min; 95 °C for 1 min; 16 cycles of 94 °C for 15 s, 63 °C for 30 s, 68 °C for 10 min, and, finally, 68 °C for 5 min. Library products were purified twice using 0.6× Ampure XP beads, and 1 μg of product was sequenced on an Oxford Nanopore PromethION 48 (Oxford Nanopore Technologies; R10.4.1).

### dscNanoCUT

First 100,000–200,000 fresh cells were centrifuged at 300× *g* for 5 min at 4 °C. We resuspended the cells in 1 mL of PBS buffer, added 12.6 μL of 16% formaldehyde (Thermo Fisher Scientific, Cat# 28906; final 0.2%), and then incubated the cells on ice for 5 min. The formaldehyde was quenched by the addition of 200 μL of 1% BSA-PBS, after which the cells were incubated on ice again for 5 min. After the supernatant was discarded, the cell pellets were first washed with 500 μL of wash buffer (20 mM HEPES, pH 7.5; 150 mM NaCl; 0.5 mM spermidine (Thermo Fisher Scientific, Cat# A19096.06), and 1× proteinase inhibitor (Roche, Cat# 11873580001) and centrifuged at 500× *g* for 5 min. The cells were resuspended in 100 μL of antibody buffer (20 mM HEPES, pH 7.5 (Gibco, Cat# 15630106), 150 mM NaCl, 0.5 mM spermidine, 2 mM EDTA, 0.01% digitonin, 0.01% IGEPAL CA-630, 0.1% BSA and 1× proteinase inhibitor) supplemented with 1:100 diluted primary antibody (anti-H3K27ac: Abcam, ab4729; anti-H3K27me3: Cell Signaling Technology, 9733S) in 200 μL PCR tubes and rotated at 4 °C for ~8 h. Then, the cells were centrifuged at 300× *g* for 3 min and washed once with 100 μL of Dig-wash buffer (wash buffer containing 0.01% digitonin and 1× proteinase inhibitor). Subsequently, the cells were resuspended with 100 μL of Dig-wash buffer with 1:50 diluted secondary antibody (donkey anti-rabbit IgG H&L antibody; Vazyme, Cat# Ab207-01) and rotated at 4 °C for 30 min. The cells were subsequently centrifuged at 600× *g* for 3 min and then washed three times with 100 μL of Dig-wash buffer. Afterward, the cells were immediately resuspended in 100 μL of Dig300-wash buffer (Dig-wash buffer containing 300 mM NaCl) with 1:100 diluted pG-Tn5 mix (Vazyme, Cat# S602) with the same adapter and rotated at 4 °C for 1 h. After pG-Tn5 binding, the cells were centrifuged at 300× *g* for 3 min, washed 3 times with 100 μL of Dig300-wash buffer, and resuspended in 100 μL of fresh tagmentation buffer (Dig-wash buffer containing 300 mM NaCl and 10 mM MgCl_2_). Then, tagmentation was performed at 37 °C for 1 h. To stop the reaction, 100 μL of stop buffer (200 μL of Dig300 wash buffer mixed with 10 μL of 0.5 M EDTA per sample) was added, followed by further incubation at room temperature for 10 min after thorough mixing. The details of the subsequent process are similar to those for dscNanoATAC.

dscNanoATAC and dscNanoCUT could also be easily applied to other droplet-based microfluidic platforms by redesigning the Tn5 adapter and amplification primer; in this study, we tested them only with the MobiCube microfluidic platform.

### dscNanoATAC/dscNanoCUT data processing

Nanoplexer (v0.1, https://github.com/hanyue36/nanoplexer) was used to divide the Nanopore sequencing reads for each library. A workflow built on the Snakemake (v5.26.1, https://snakemake.readthedocs.io) framework was subsequently developed to demultiplex and preprocess the single-cell data. Specifically, to increase computational efficiency, the raw FASTQ files were first split into chunks using seqkit^51^ (https://bioinf.shenwei.me/seqkit) with the command “seqkit split2 -s {params.size} -t dna -j {threads} -1 {input} -O {output}” for parallel processing. For each chunk, the adapter sequences were trimmed with cutadapt (v3.3, https://cutadapt.readthedocs.io/) with the additional parameters “-q 7 -e 0.25 -m 100”. Two rounds of barcode extraction were then performed for both ends of the reads with umi_tools^19^. In detail, we first used the command “umi_tools extract” in regex mode at the 5’ ends of the reads with the following barcode pattern:

“(?P < cell_1 > .{10}){e ≤ 2}(?P < discard_1 > .{0,3})(?P < discard_2 > TCGAG){e ≤ 1}(?P <cell_2 > .{10}){e ≤ 2}(?P<umi_1 > .{10})(?P < discard_3 > CACCGTCTCCGCCTCAGATGTGTATAAGAGACAG){e ≤ 7}.*”

which sets an error rate of ~0.2 to match the two 10-bp cell barcodes and anchor sequences in each read and then appends the detected barcodes to their read names. A manually curated whitelist containing the full combination of barcode1 and barcode2 was used to filter invalid barcodes derived from sequencing errors. Read groups both with and without identified valid barcodes were output; they were then converted to their reverse complementary sequences with “seqkit seq -r -p {input}”. Afterward, a second run of barcode extraction at the original 3’ end was performed. Reads that failed to match the barcodes within the provided whitelist or that harbored different barcodes on opposite ends were filtered. The retained reads were mapped to the genome (hg38 or mm10) using minimap2^52^ (v2.18-r1015, https://github.com/lh3/minimap2) with the parameters “-ax map-ont --secondary=no”. For cross-species contamination experiments, the hg38 and mm10 genomes were concatenated to generate a mixed genome. After alignment, cell barcodes in the read name were manually added to the “CB” tag of the BAM files. Samtools was further used to retain primary alignments and remove alignments with long clipping sequences (as these may cause shifting of the true Tn5 insertion sites and introduce biases) using the command “samtools view -ShuF 2308 -q 30 -e “sclen < 150 & & hclen < 150” {input}”. Finally, the filtered bam files from each chunk were gathered and deduplicated using “umi_tools dedup”^14^ with the following parameters: “--extract-umi-method=tag --per-cell --ignore-umi --cell-tag=CB --temp-dir=mapping -I {input} -L {log} -S {output}”, the resulting “clean” bam file was converted to fragments using the “bamtobed” command of bedtools with default parameters.

### Dimensionality reduction and clustering

We followed the ArchR^20^ workflow to perform basic single-cell analysis. Specifically, ArchR (v1.0.1) was used to create arrow files for each sequencing library because of its compatibility with the long fragment length of the dscNanoATAC/dscNanoCUT data (in future versions, fragments longer than 1000 bp will be discarded). Once the arrow files were created, the number of unique fragments in each cell barcode was summarized, and the threshold for identifying the true cell barcodes was determined by estimating the local minima of the distribution of the log_10_-scaled fragment counts per cell barcode, as described by Smith et al.^19^. Cell barcodes that contained more fragments than the identified threshold were retained and further filtered according to the following thresholds: number of unique fragments > 1000 and TSS enrichment score > 1. Afterward, downstream analyses were performed with ArchR (v1.0.2). We removed potential doublets on the basis of the synthetic doublet strategy implemented in ArchR. For dimensionality reduction, two rounds of iterative LSI were performed with the “addIterativeLSI” function using a tile matrix with default parameters, following UMAP and Louvain community detection to obtain clusters via the “addUMAP” and “addClusters” functions of ArchR.

For the mouse brain datasets, we used Signac^53^ (v1.14.0) for dimensionality reduction and clustering, as we found that the clustering workflow of this program was more sensitive, allowing us to identify the neuronal cell subtypes. In detail, we first converted the fragment files into Tn5 insertion counts by taking 1 bp from both ends of the fragments. Afterward, cell-by-bin matrices were constructed using 5-kb non-overlapping genomic bins. We used the top 85% of the genomic bins to perform term frequency-inverse document frequency and singular value decomposition. The batch effects were corrected using Harmony with default parameters and used for UMAP visualization and Louvain clustering.

### Peak calling and motif enrichment

To call the peaks for each cluster, we first generated the aggregated pseudo-bulk replicates of single cells using the “addGroupCoverages” command of ArchR, in which the “minCells” and “maxCells” parameters were adjusted on the basis of the number of cells in each cluster, to enable the use of all cells. Afterward, the peaks were called using MACS2 via the “addReproduciblePeakSet” function of ArchR with the parameters “cutoff = 0.05, reproducibility = 2, groupBy = “Clusters”, and additionalParams = “--nomodel --llocal 500000””, which generated fixed-width peak sets for each pseudo-bulk replicate, and merged with an iterative overlapping strategy. Differentially accessible/occupation peaks were then identified between clusters using Wilcoxon’s rank-sum test by accounting for the biases introduced by the TSS enrichment score and log_10_(nFrags). Significant peaks were selected with the parameters “FDR ≤ 0.05 & Log_2_FC ≥ 0.5”. Motif enrichment was performed using the “peakAnnoEnrichment” function against the motifs of transcription factors derived from the CISBP, ENCODE, and HOMER databases with default parameters.

### Identification of allele-specific peaks

To identify allele-specific peaks, the aligned BAM files were haplotagged by whatshap (v1.3, https://whatshap.readthedocs.io/) on the basis of known SNP sites with the command “whatshap haplotag -o {output} --reference {input.ref} --ignore-read-groups --tag-supplementary --skip-missing-contigs {input.snp} {input.bam}”. For GM12878, the SNP sites were downloaded in VCF format from the Illumina Platinum Genome website (https://github.com/Illumina/PlatinumGenomes, hg38, 2017-1.0 version). The SNP sites of DBA/2 and C57BL/6 mouse strains were downloaded from MGP website (MGPv5, dbSNP142), and only homozygous SNPs of DBA/2 that were heterozygous with those of C57BL/6 were used for haplotype tagging. Afterward, the tagged BAM files were split using samtools on the basis of the values of the “HP” tags and converted to fragment files using the “bamtobed” command of bedtools. We subsequently counted the number of paternal and maternal fragment ends located in each peak of each cluster. A two-sided exact binomial test was used to determine whether there was significant bias toward paternal or maternal counts. We also calculated the ratio of paternal fragments to total allelic-specific fragments for each peak; peaks that passed the binomial test (*P* value < 0.05) with a paternal ratio > 0.5 were identified as paternal-specific peaks; otherwise, they were identified as maternal-specific peaks.

### Identification of co-accessibility events

The long-read characteristic of dscNanoATAC allows us to identify the physical links between nearby peaks and thus determine their co-accessibility. We used a previously described method with minor modifications to identify these events^16^. In brief, if two peaks were linked by multiple fragments, the distribution of the lengths of these fragments will be narrow and close to the distance between the two peaks. In practice, the length distributions of supporting fragments (those whose ends fall into a 1 kb region centered on the peak of interest) and background fragments (100-kb region) on each side of each peak were calculated. The Kolmogorov‒Smirnov test was used to determine whether there was a significant difference between the lengths of the supporting fragments and those of the background fragments. Afterward, neighboring significant peaks in opposite directions were paired as co-accessibility candidates and further filtered on the basis of the fold change between their average fragment length and peak distance; given the assumption that the length distribution of supporting fragments should be centered at the peak distance, only peak pairs with a fold change < 2 and > 0.5 were selected as the final co-accessibility events.

### Analysis of peaks in low-mappability regions

To determine the advantages of long reads in detecting signals in complex genomic regions, the mappability of 150 bp and 3 kb reads in both the hg38 and mm10 genome assemblies was calculated with GenMap^54^ (v1.3.0, https://github.com/cpockrandt/genmap). Genomic regions whose average mappability was less than 0.5 were defined as low-mappability regions. The low-mappability regions in the 150 bp reads that showed higher mappability in the 3 kb reads should represent regions with improved mappability when long sequencing reads are used. We also calculated the average mappability of repeat elements on the basis of the annotations derived from RepeatMasker and analyzed their overlaps with peaks derived from our methods using the R package “gUtils” (https://github.com/mskilab-org/gUtils).

### Identification of cCRE modules

We used previously described methods^13^ to identify the cCRE modules for the dscNanoATAC and dscNanoCUT mouse brain datasets. Specifically, we first calculated the average signal levels for each cell type and normalized them to the RPKM values. Only cCREs with a signal RPKM value > 1 and detected in more than 3 (dscNanoCUT) or 5 cells (dscNanoATAC) were kept for downstream analyses. We then classified cCREs as proximal or distal on the basis of their relative location to promoters (± 2 kb of the TSS). Afterward, NMF was performed to group the cCREs into modules. We also subjected the cCRE modules to GO enrichment analysis using the R package rGREAT (v2.5.7)^55^.

### Prediction of allele-specific 3D genome structures

We performed cell type-specific 3D genome structure prediction on the basis of the C.Origami architecture, which leverages DNA sequence, cell type-specific ATAC-seq, and CTCF ChIP-seq signals to predict 3D genome structures with a deep learning model^31^. To accurately predict the structure of GM12878, we trained a C.Origami model de novo using the ATAC-seq, CTCF ChIP-seq, and Hi-C data of GM12878 provided in https://zenodo.org/record/7226561 with the recommended parameters. Afterward, the allele-specific structures were predicted by using the allele-specific ATAC-seq signals generated by dscNanoATAC as inputs to the GM12878 C.Origami model. To evaluate the prediction results, we also collected deep-sequenced in situ Hi-C data from the 4DN website^32^ (4DNES3JX38V5) and haplotagged them with whatshap (v1.3, https://whatshap.readthedocs.io/en/latest/) to generate allele-specific Hi-C contact maps. The allele-specific Pore-C data phased by dip3d were also included in the benchmarking step^34^. Given the sparsity of SNP sites and the resulting contact maps, we next calculated the 10-kb SNP coverage for each 2-Mb window with 500-kb steps across the genome and selected the top 100 windows as candidate regions for performing benchmark analysis between the predicted and observed allele-specific contact maps. The HiCRep^33^ package was used to calculate the paired SCCs. Since the observed maps were much sparser than the predicted maps, which may result in systematic differences, we then calculated the SCCs between the paternal and maternal contact maps in the observed and predicted results separately and calculated a second correlation coefficient for the SCC to evaluate the consistency between the predicted and observed results. We also calculated the insulation scores using a sliding window-based method (200-kb windows, 10-kb resolution), similar to the implementation of cooltools^56^, and compared them between the observed and predicted contact maps.

## Supplementary information

Supplementary Information

Supplementary Table S1. metadata of dscNanoATAC and dscNanoCUT

Supplementary Table S2. Allele-specific accessible peaks in GM12878 identified by dscNanoATAC

Supplementary Table S3. Neighboring co-accessibility in GM12878 identified by dscNanoATAC

Supplementary Table S4. GM12878 accessible peaks located in low-mappability regions

Supplementary Table S5. dscNanoATAC cCRE modules in mouse brain

Supplementary Table S6. Allele-specific accessible peaks of mouse brain

Supplementary Table S7. Neighboring co-accessibility in mouse brain

Supplementary Table S8. Acessible peaks of mouse brain located in low-mappability regions

Supplementary Table S9. Allele-specific H3K27ac peaks in GM12878 identified by dscNanoCUT

Supplementary Table S10. dscNanoCUT cCRE modules in mouse brain

Supplementary Table S11. TE copies with H3K27ac or H3K27me3 modifications in mouse brain

Supplementary Table S12. Primer information of dscNanoATAC and dscNanoCUT

## Data Availability

The sequencing data of dscNanoATAC and dscNanoCUT have been deposited in the Gene Expression Omnibus under accession code GSE293463.

## Code availability

Code for dscNanoSeq pipeline is available in GitHub (https://github.com/LuJiansen/dscNanoSeq) and Zenodo (https://doi.org/10.5281/zenodo.15102690)^57^. Code for AS-C.Origami is deposited in GitHub (https://github.com/LuJiansen/AS-C.Origami) and Zenodo (https://doi.org/10.5281/zenodo.21389362)^58^.
