# Supplementary material for: A droplet-based long-read single-cell epigenome sequencing method for the high-throughput profiling of multidimensional regulatory elements within individual cells
Source: Cell Discov. 2026 Oct 27;12:72. doi: 10.1038/s41421-026-00928-y (PMC13710847; doi:10.1038/s41421-026-00928-y)
Supplement: Supplementary file 1 [file 41421_2026_928_MOESM1_ESM.pdf]

Supplementary Figures

Supplementary Fig. S1

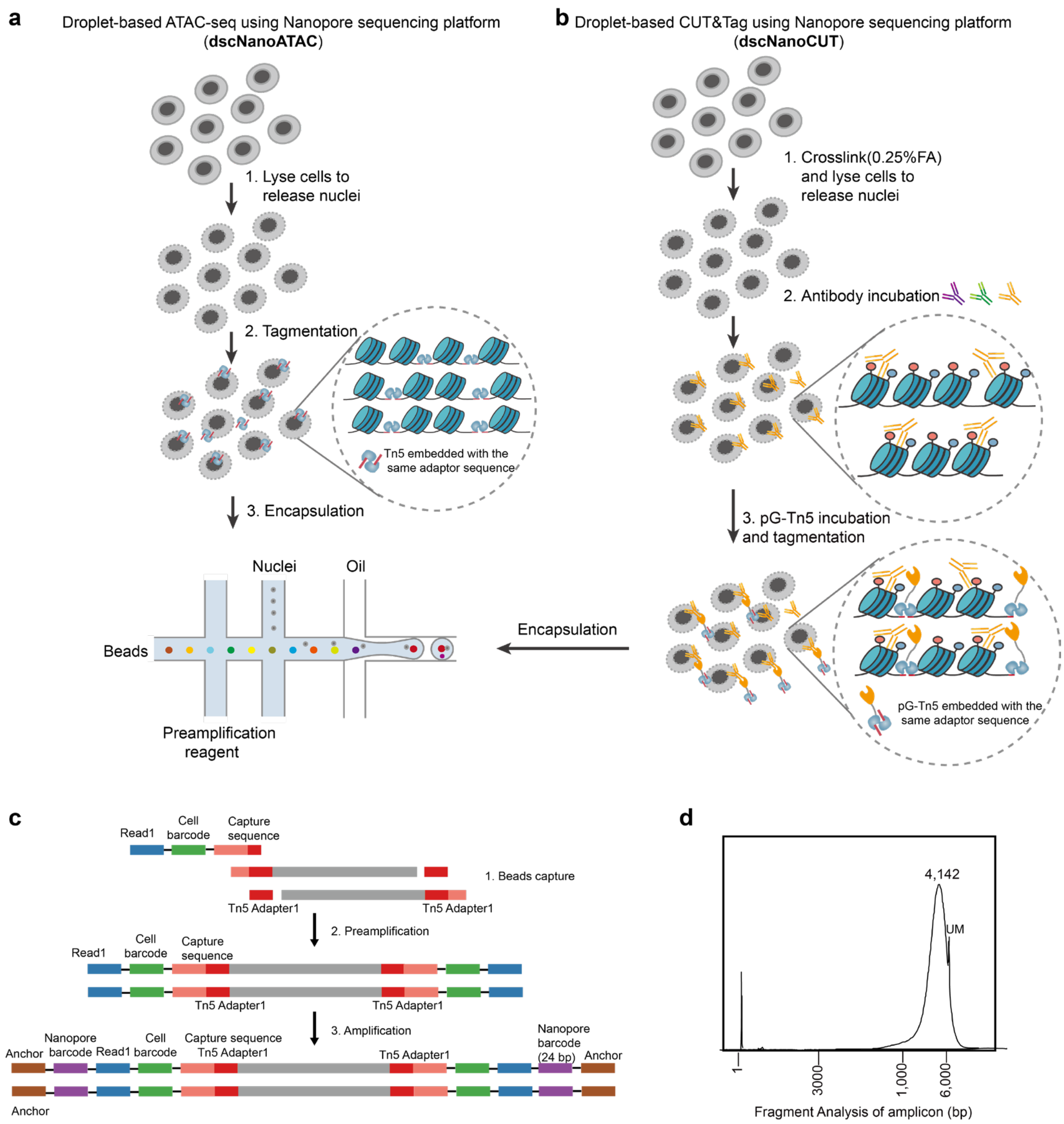

Supplementary Fig. S1 The detailed workflow of dscNanoATAC and dscNanoCUT. a. The workflow of dscNanoATAC. **b.** The workflow of dscNanoCUT. **c.** The library structure of dscNanoATAC and dscNanoCUT. **d.** The length distribution of the final amplicon of dscNanoATAC and dscNanoCUT.

Supplementary Fig. S2

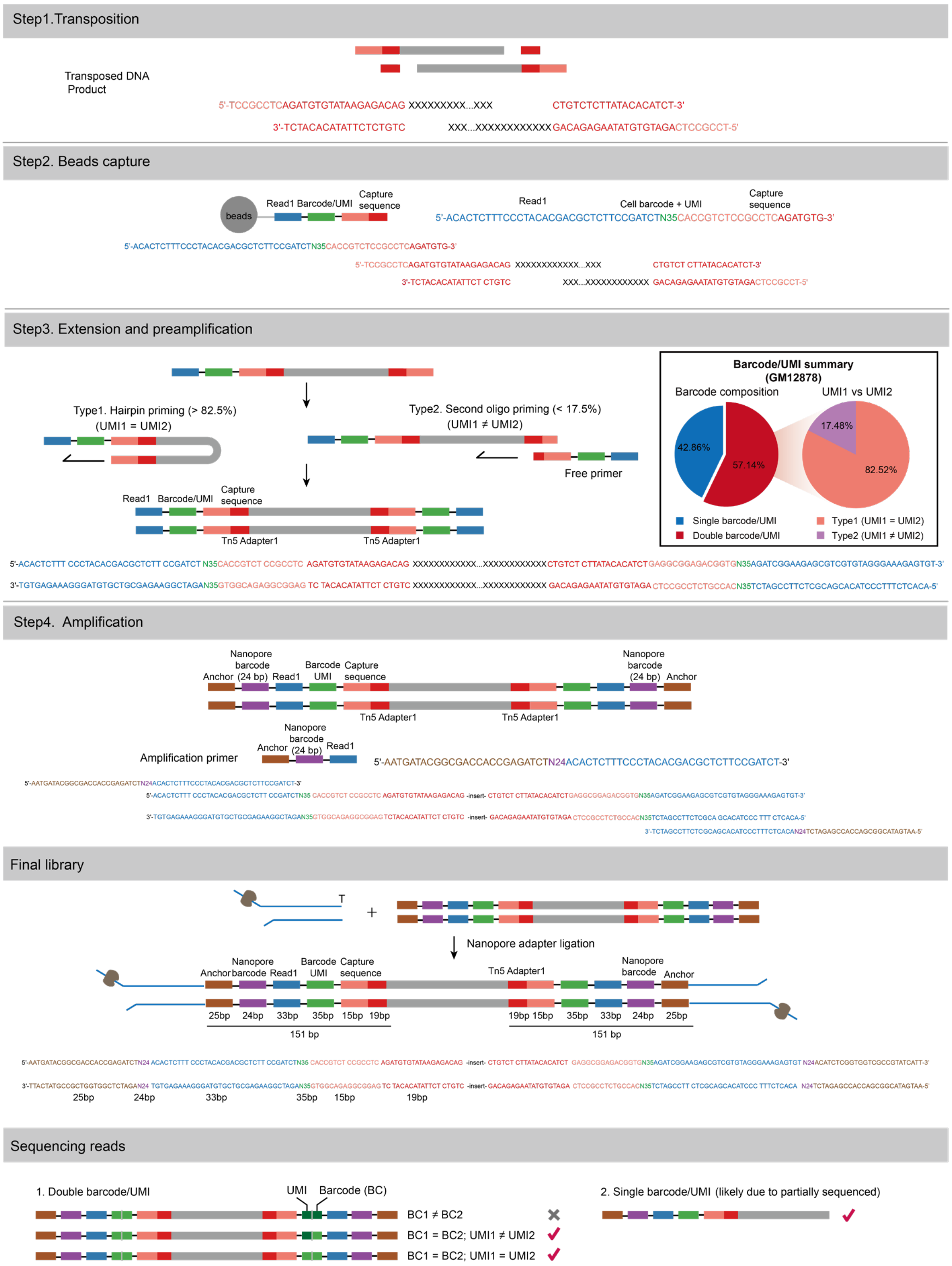

Supplementary Fig. S2 Primer design and amplification scheme of dscNanoATAC. The genome was first transposed by mono-adapter Tn5, then captured by bead oligos in the microfluidic platform. In the extension and pre-amplification step, there are two possible mechanisms for 3' priming. Pie charts on the right show the barcode composition of the GM12878 sample and the percentage of dual-barcoded reads containing identical UMIs. After that, product was amplified by a single primer containing Read1 and ligated to Nanopore sequencing adapters before sequencing on Nanopore.

Supplementary Fig. S3

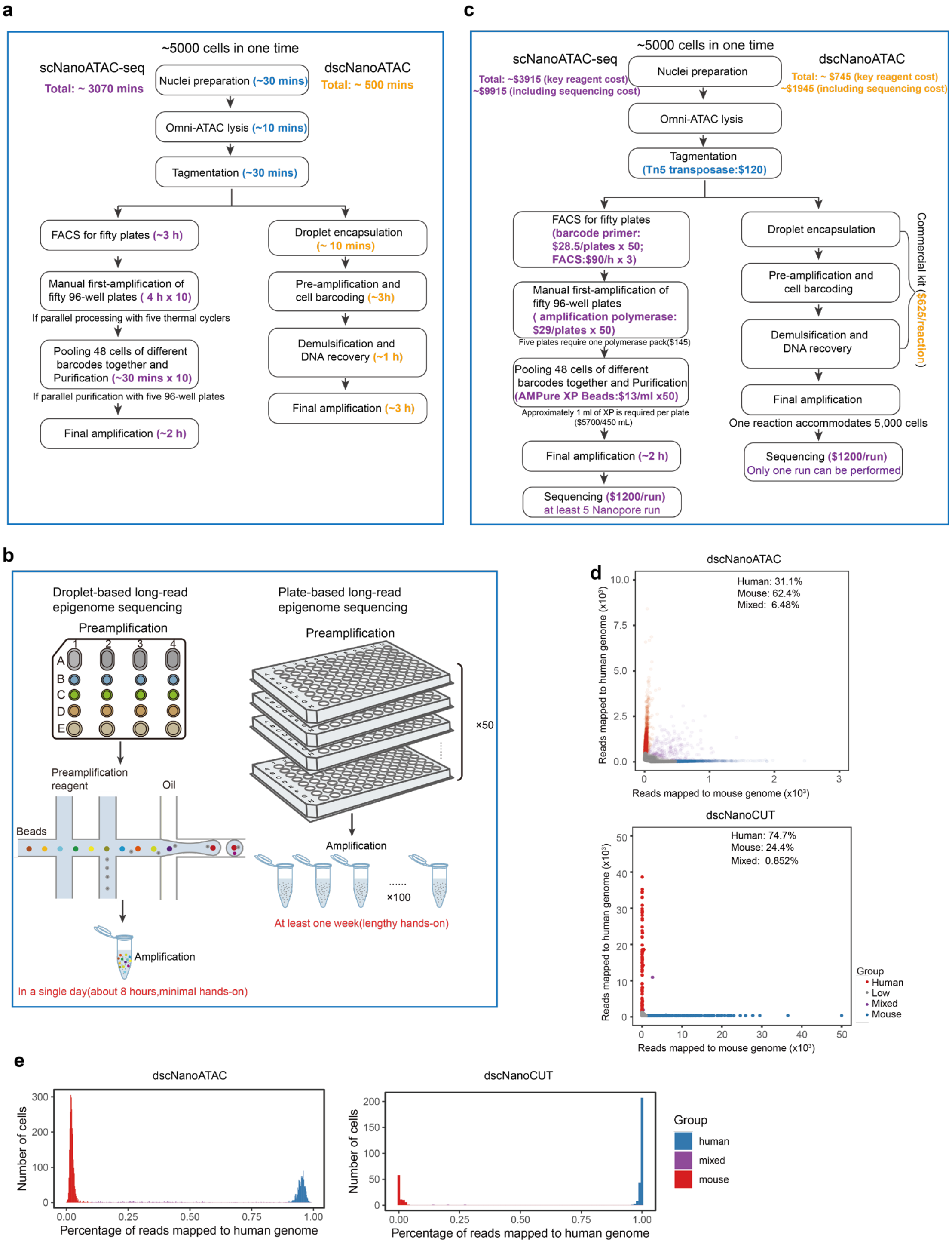

Supplementary Fig. S3 Comparison between droplet-based and plate-based single-cell long-read epigenome sequencing methods. a. Comparison of specific experimental operation time between dscNanoATAC and scNanoATAC-seq at a throughput of 5,000 cells. **b.** Comparison of workflow between dscNanoATAC and scNanoATAC-seq at a throughput of 5,000 cells. **c.** Comparison of specific experimental time between dscNanoATAC and scNanoATAC-seq at a throughput of 5,000 cells. **d.** Scatter plot indicating the collision rates of the dscNanoATAC (top) and dscNanoCUT (bottom), respectively. **e.** Histogram showing the percentage of reads mapping to the human genome in each cell from dscNanoATAC (left) and dscNanoCUT (right).

Supplementary Fig. S4

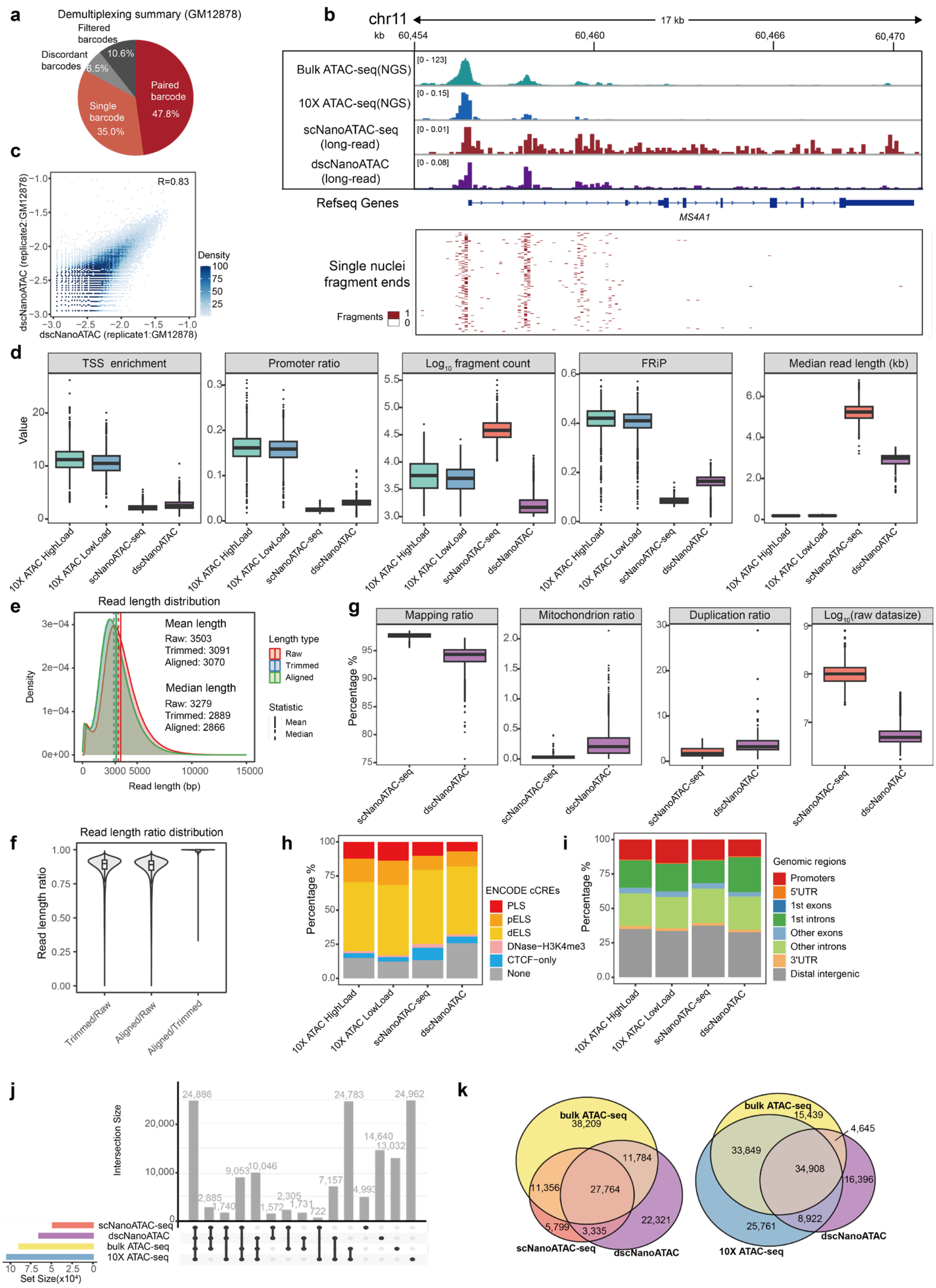

Supplementary Fig. S4 The quality control of dscNanoATAC data in the GM12878 cell line. a. The demultiplexing summary of sequencing data for GM12878. **b.** Genome browser view showing examples of pseudo-bulk and single-cell chromatin accessibility signals of dscNanoATAC in GM12878, along with bulk ATAC-seq, 10X scATAC-seq, and scNanoATAC-seq. The associated gene, *MS4A1*, is a specific marker gene of B cells. **c.** The reproducibility of dscNanoATAC. Scatter plots illustrating the Pearson's correlation coefficients of genome-wide chromatin accessibility

between two independent biological replicates of GM12878. **d.** Quality control metrics, including TSS enrichment, promoter ratio, number of fragments (Log_{10}-scaled), FRiP (Fraction of Reads in Peaks), and median read length identified by dscNanoATAC, high- and low-loading 10X scATAC-seq, and scNanoATAC-seq. **e.** Read length distribution during data preprocessing. **f.** Read length ratio during data preprocessing. **g.** The mapping ratio, percentage of fragments from mitochondria (mitochondrion ratio), duplication ratio, and raw data size of dscNanoATAC and scNanoATAC. **h-i.** Distribution of chromatin accessible peaks across candidate cis-regulatory elements (cCREs) (**h**) and gene regions (**i**) detected by dscNanoATAC, high- and low-loading 10X scATAC-seq and scNanoATAC-seq in GM12878. **j-k.** Upset plot (**j**) and Venn diagrams(**k**) showing the overlaps among chromatin accessible peaks identified by dscNanoATAC, bulk ATAC-seq, 10X scATAC-seq, and scNanoATAC-seq in GM12878 cells.

Supplementary Fig. S5

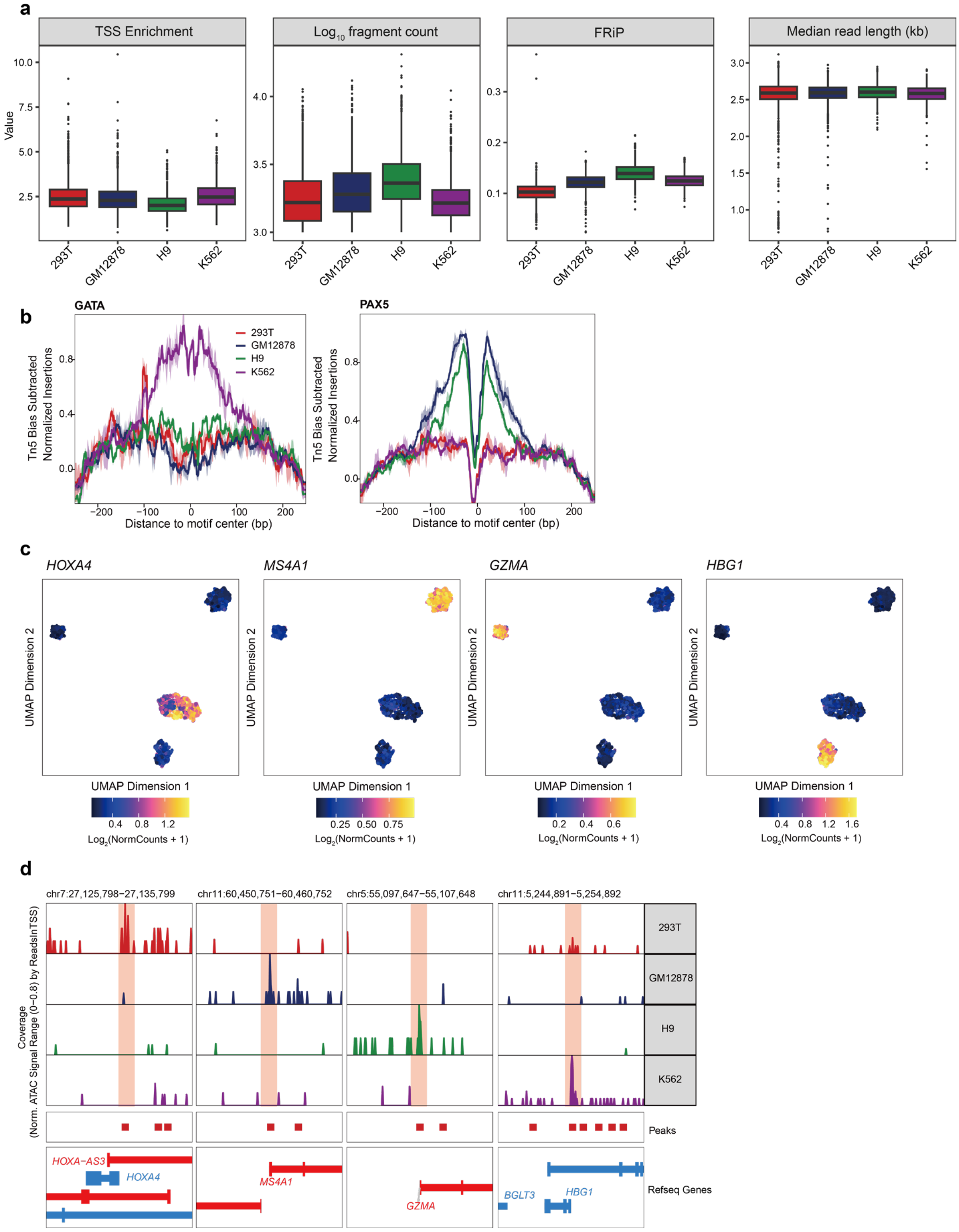

Supplementary Fig. S5 The quality control of dscNanoATAC data in four cell lines. a. Quality control metrics of scNanoATAC-seq, including TSS enrichment, number of fragments (Log_{10}-scaled), FRiP (Fraction of Reads in Peaks), and median read length in four cell lines (GM12878, K562, 293T, and H9). **b.** Footprints of K562-specific TF GATA1 and GM12878-specific TF PAX5. **c.** Gene scores of marker genes visualized on UMAP embedding. **d.** Track view of marker genes in four cell lines, from left to right are marker genes of 293T, GM12878, H9, and K562 cell lines, respectively.

Supplementary Fig. S6

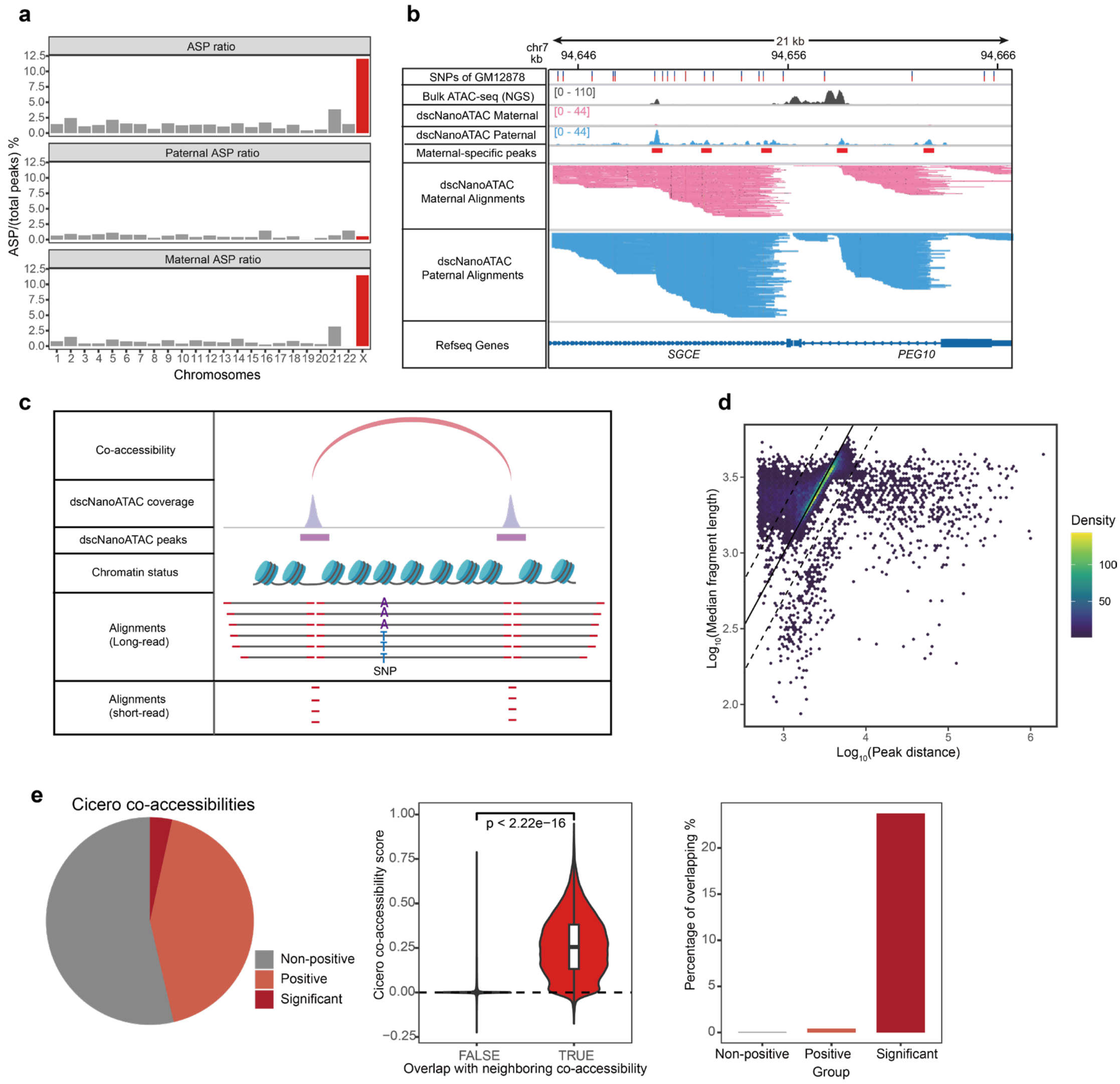

Supplementary Fig. S6 Allele-specific peaks identified by dscNanoATAC data. a. Proportions of ASPs in GM12878 distributed across each chromosome. **b.** Genome browser view of representative ASPs at *SGCE* locus, from top to bottom are SNP sites of GM12878, ATAC signals of bulk ATAC-seq, maternal and paternal dscNanoATAC, maternal-specific peaks identified by dscNanoATAC, maternal and paternal alignments from dscNanoATAC and RefGene annotations, respectively. **c.** Schematic diagram illustrating the identification of co-accessible peaks with dscNanoATAC. **d.** Scatter plots indicating the filtration of co-accessibility events. **e.** The proportion of Non-positive, positive, and significant co-accessibilities detected by Cicero (left); the Cicero co-accessible scores of Cicero co-accessibilities overlapping or non-overlapping with neighboring co-accessibilities (middle); the percentage of co-accessibilities overlapping with neighboring co-accessibilities in the three Cicero groups.

Supplementary Fig. S7

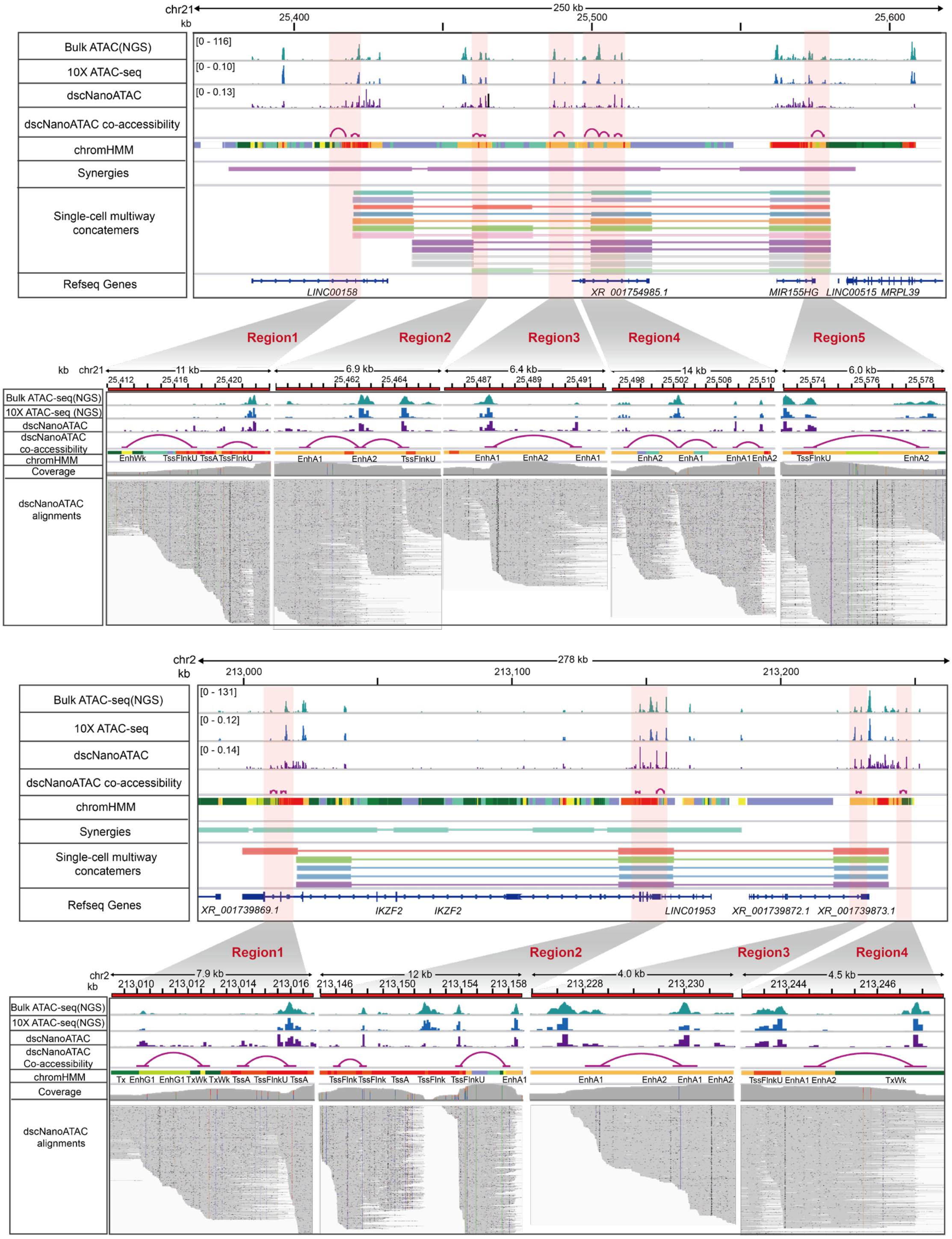

Supplementary Fig. S7 Relationships between neighboring co-accessibility and multiway interactions. Genome browser view showing multiple chromatin co-accessibility events at *MIR155HG* and *IKZF2* locus, which correspond to synergies and multiway concatemers (high-order promoter-enhancer interactions) predicted by scNanoHi-C[18]. The red shadow highlights the co-accessibility region inside *MIR155HG* and *IKZF2*. The associated genes include *MIR155HG*, an EBV super-enhancer-associated gene, and *IKZF2*, which encodes a B-cell-specific transcription factor (TF).

Supplementary Fig. S8

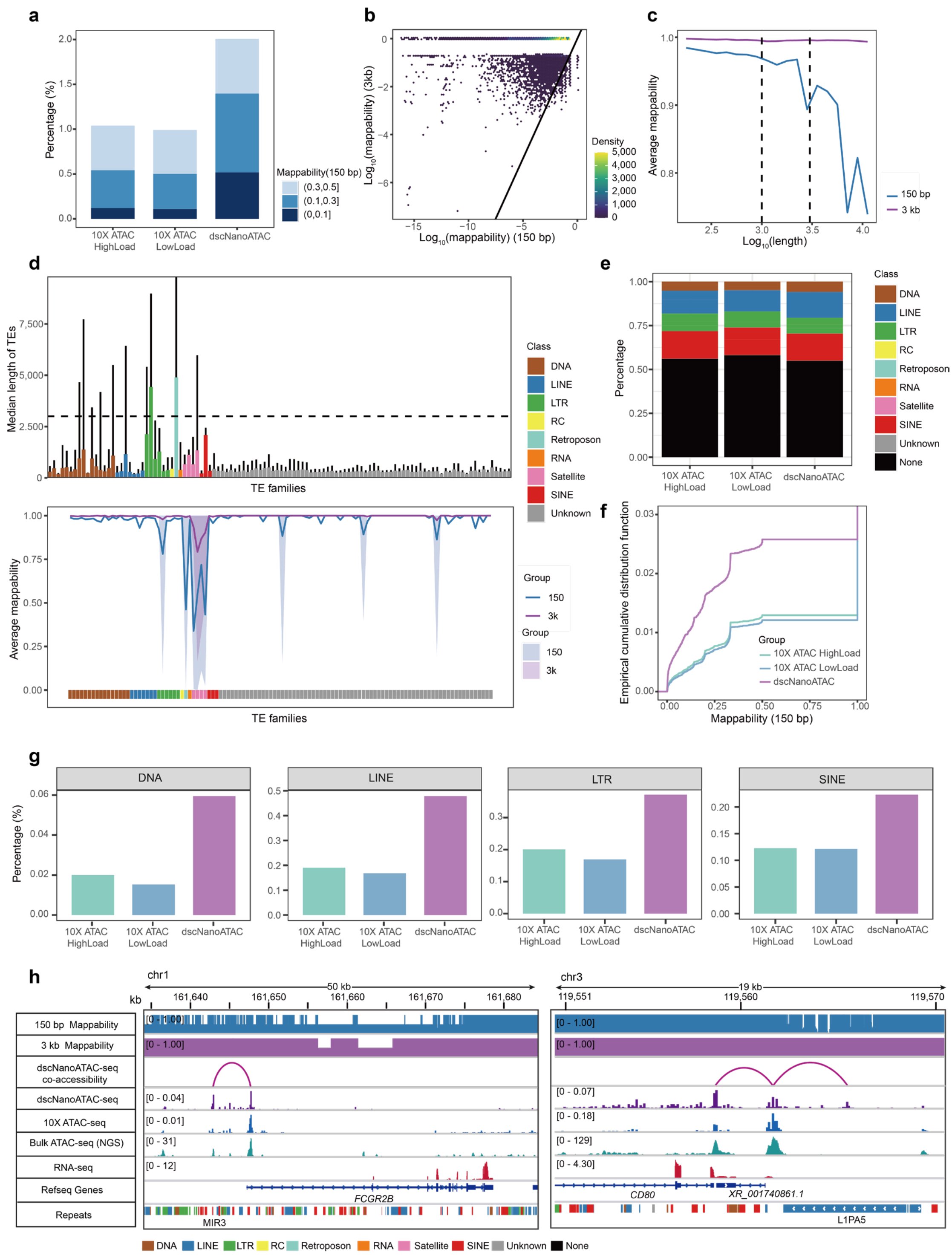

Supplementary Fig. S8 TE elements identified by dscNanoATAC data. a. The percentage of mappability evaluated by high- and low-loading 10X scATAC-seq and dscNanoATAC. **b.** The distribution of mappabilities (150 bp and 3 kb) on all copies of repeat elements. **c.** The relationship between the length of repeat elements and their average mappabilities in 150 bp and 3 kb. **d.** Median length (top) and average mappability of TE families detected by 150 bp and 3 kb reads (bottom). **e.** The percentage of peaks overlapping with different TE families detected by high- and low-loading

10X scATAC-seq and dscNanoATAC. **f.** Empirical cumulative distribution of the mappabilities of identified peaks using high- and low-loading 10X scATAC-seq and dscNanoATAC, indicating the percentage of peaks located in low-mappability regions (mappability < 0.5). **g.** The percentage of peaks located in repeat elements of DNA, LINE, LTR, and SINE classes detected by high- and low-loading 10X scATAC-seq and dscNanoATAC. **h.** The genome browser view shows the representative regions containing the accessible signals at repeat elements, including the MIR3 elements upstream of *FCGR2B*, which also exhibit co-accessibility with the *FCGR2B* promoter (left), and the accessible signals at L1PA5 elements upstream of *CD80* (right).

Supplementary Fig. S9

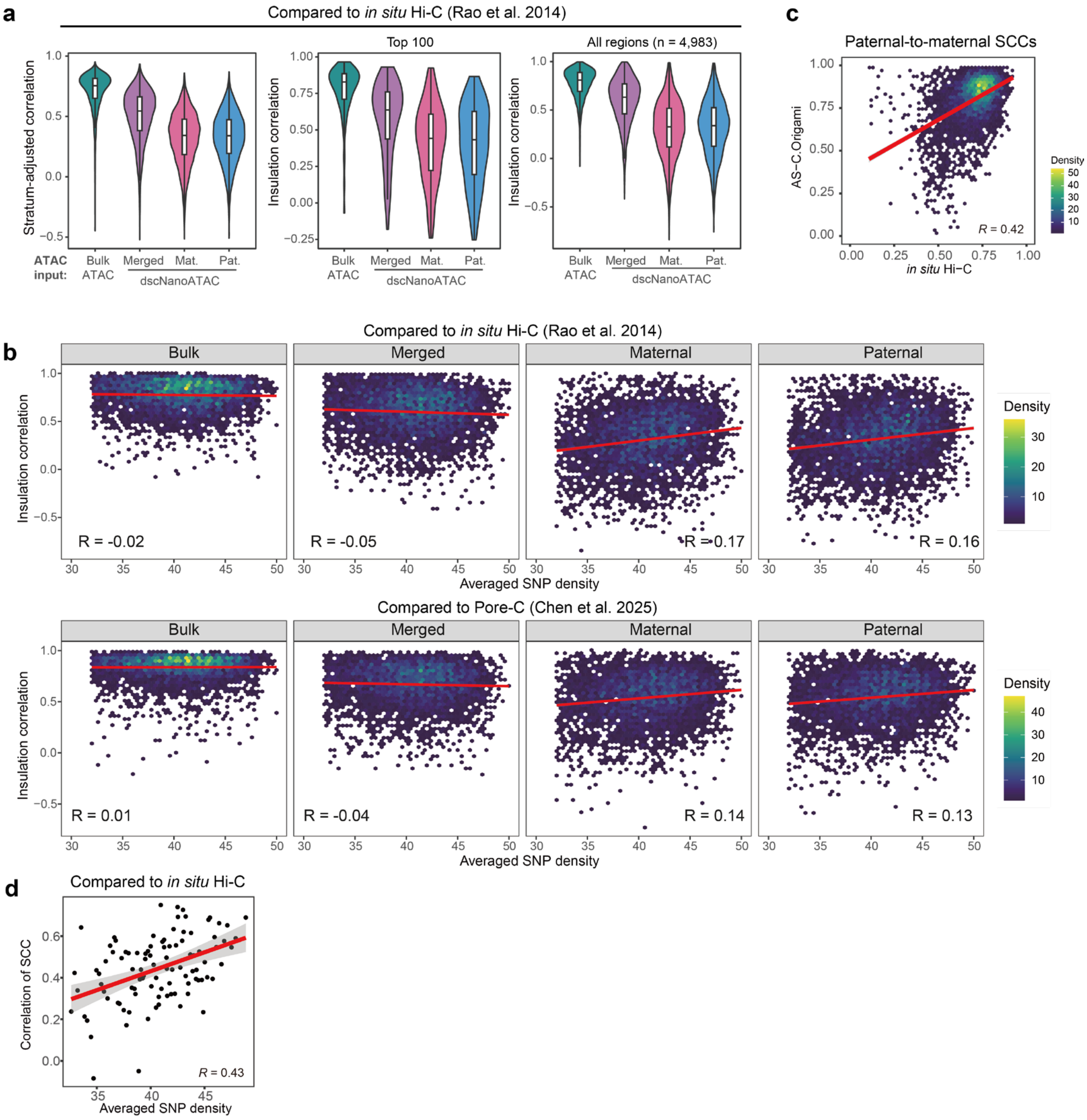

Supplementary Fig. S9 Benchmark analyses of Allele-specific C. Origami workflow. a. Stratum-adjusted correlation coefficient **(**SCC) and correlation of insulation scores between predicted and experimental results (*in situ* Hi-C). The predictions were based on bulk ATAC-seq, merged, paternal, and maternal dscNanoATAC data, respectively. **b**. The relationship between the correlation of insulation scores and SNP density, compared to *in situ* Hi-C (top) and Pore-C data (bottom). **c**. Correlation of paternal-to-maternal SCCs between AS-C.Origami and bulk Hi-C (n = 4,983). **d**. Correlation of paternal-to-maternal SCCs between AS-C.Origami and bulk Hi-C with varying SNP density. A total of 4,950 2-Mb genomic regions were sorted by SNP density, divided into groups of 50 to calculate correlations separately for each group.

Supplementary Fig. S10

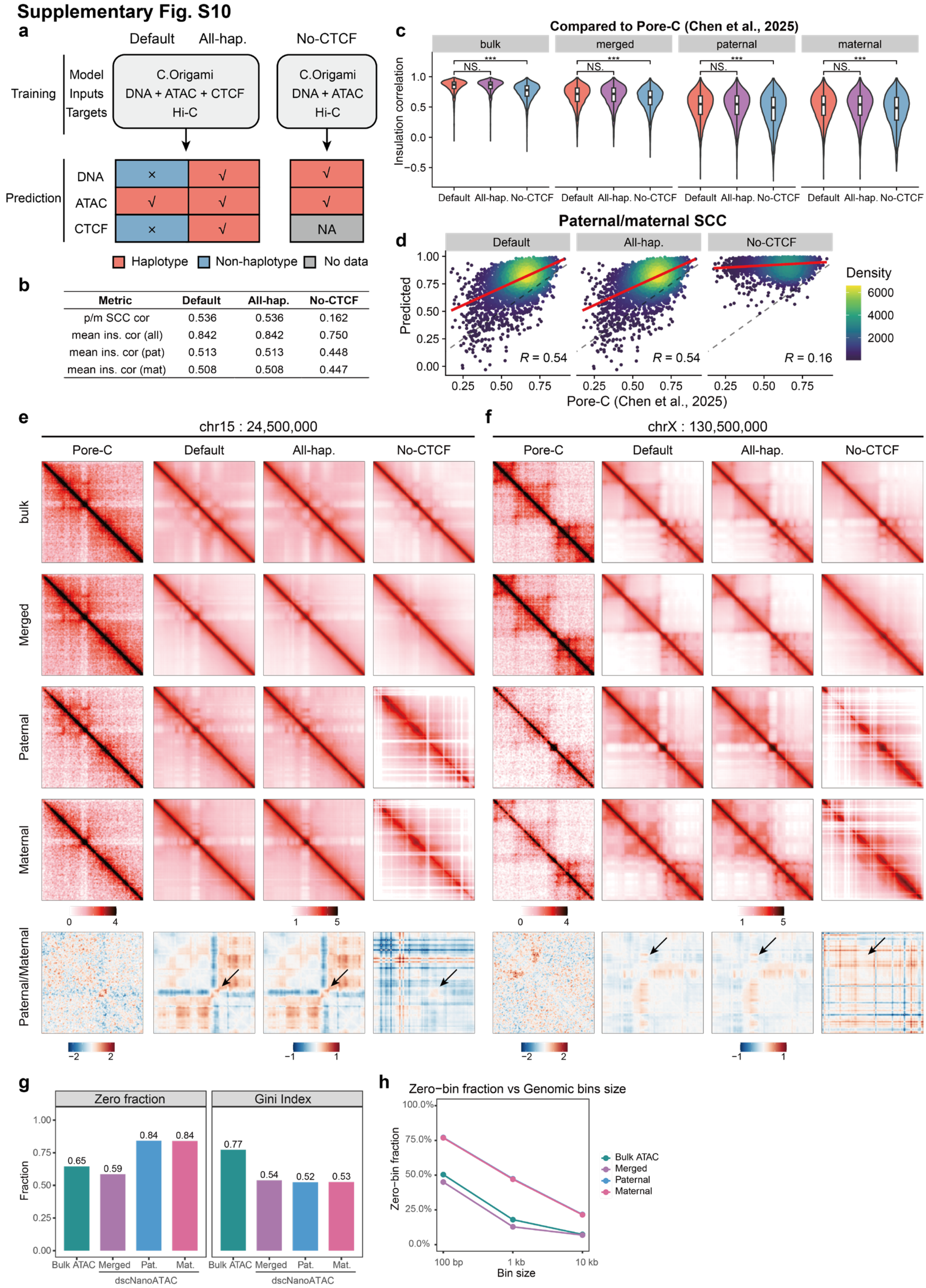

Supplementary Fig. S10 Benchmarking of different haplotype-specific Hi-C prediction strategies. a-b. The schematic of three different prediction strategies (**a**) and the summary of their benchmarking metrics (**b**). **c**. correlation of insulation scores between predicted and experimental results, calculated for merged, paternal, and maternal data, respectively, compared to Pore-C data (Chen et al. 2025). **d.** Correlation of paternal-to-maternal SCCs between predicted results and Pore-C data, Pearson's correlation coefficients are shown in the bottom right corner. **e-f**. Contact maps

of merged, paternal, maternal and paternal-to-maternal ratio of two representative regions. The differential structures between parental alleles were highlighted with arrows. **g.** The fraction of zero bases (1-bp resolution) and Gini Index of signal in the bigwig files of bulk ATAC-seq (from C.Origami dataset), merged, paternal and maternal dscNanoATAC. **h.** Multiple scaled fractions of zero bins in the bigwig files related to (**g**).

Supplementary Fig. 11

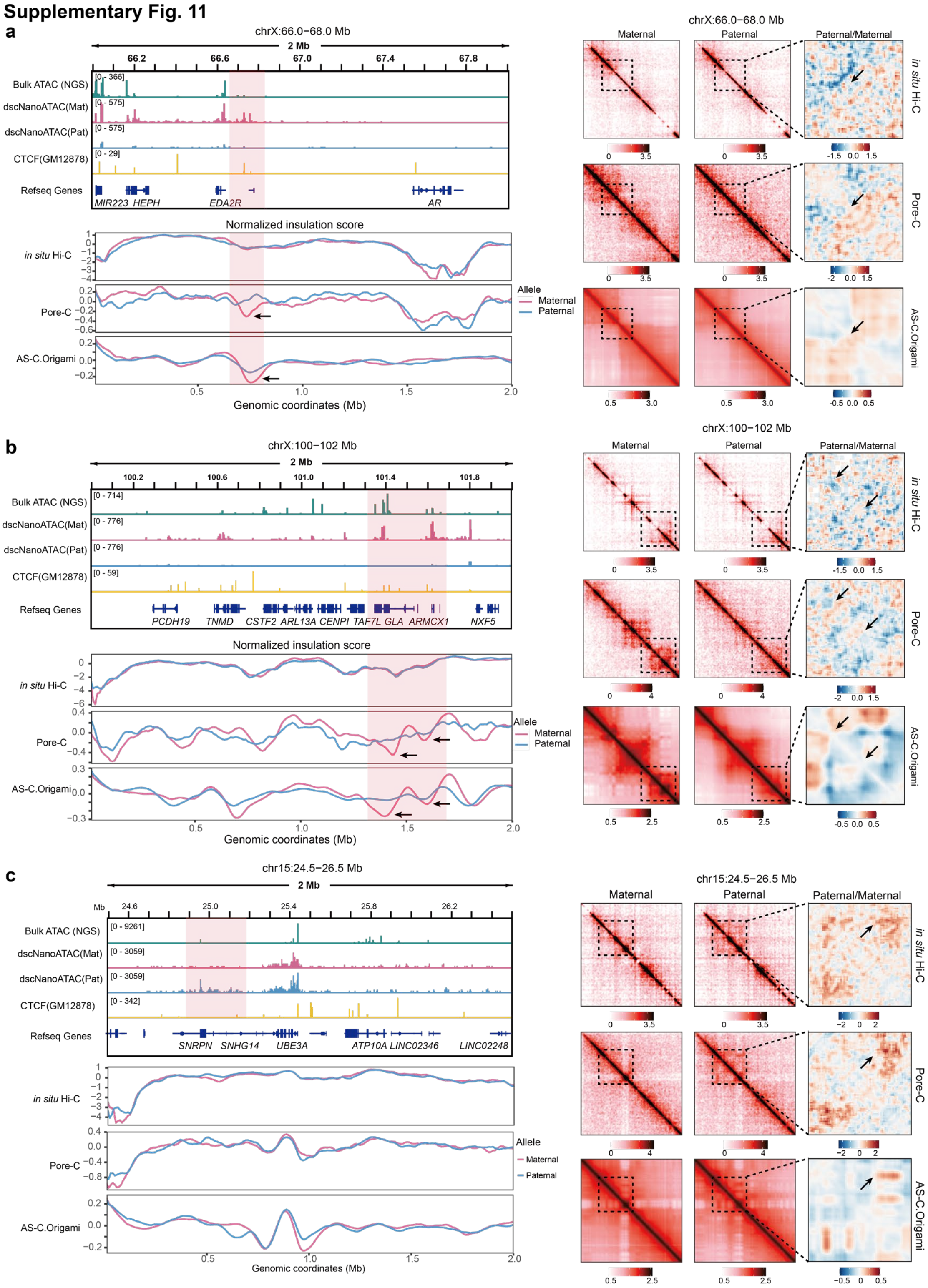

Supplementary Fig. S11 Allele-specific C. Origami predicted the allele-specific 3D structures. **a-c**. Genome browser view showing allele-specific accessibility (left top), associated insulation profiles (left bottom), and contact maps from bulk Hi-C, Pore-C, and AS-C.Origami (right). In the right panel, the merged, maternal and paternal contact map and the differential contact map between paternal and maternal allele (red and blue indicated paternal- and maternal-specific interactions, respectively) were shown. The differential structures between parental alleles were highlighted with

arrows. Two representative regions in X chromosome **(a-b)** and the *UBE3A* locus **(c)** were displayed.

Supplementary Fig. S12

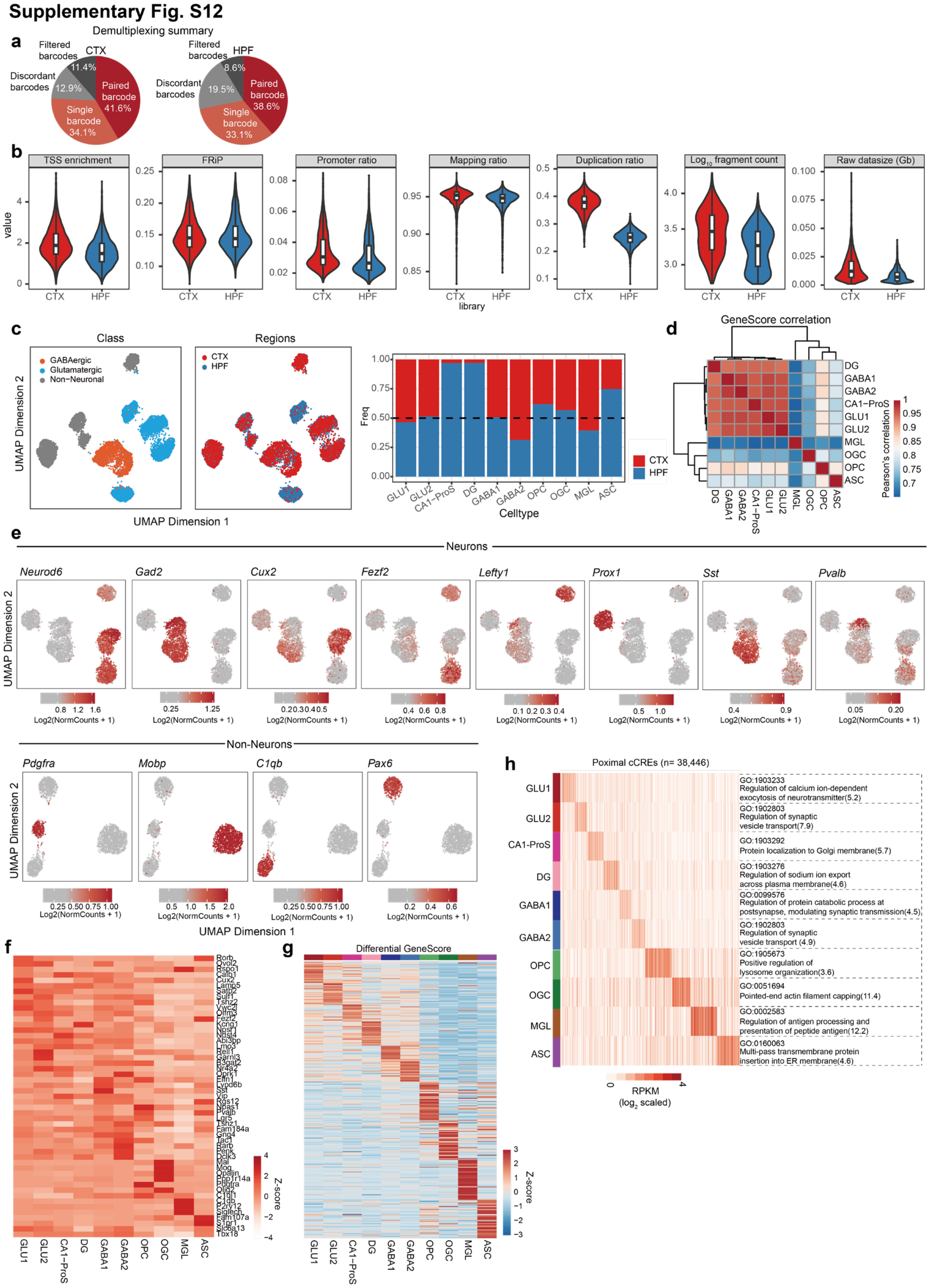

Supplementary Fig. S12 dscNanoATAC identified cell populations in the mouse brain. a. The demultiplexing summary of sequencing data for CTX and HPF in the mouse brain using dscNanoATAC. **b.** Boxplots indicating the basic quality control metrics of the mouse brain dataset, including the TSS enrichment score, FRiP, promoter ratio, mapping ratio, duplication ratio, number of fragments (Log$_{10}$-scaled), and raw data size of dscNanoATAC in adult mouse CTX and HPF. **c.**

UMAP showing the classes and regions in different cell types of the mouse brain (left); UMAP and barplot showing the distribution and percentage of cells from the cerebral cortex and hippocampus in each cluster (right). **d.** The gene score correlation between different cell types. **e.** Gene scores of marker genes in each cluster are visualized on the UMAP embedding. **f.** Heatmap showing the averaged Gene Scores of known marker genes in each cell type. **g.** Heatmap showing the averaged Gene Scores of differentially activated genes identified in each cell type. **h.** Proximal cCRE modules of each cell type in the mouse brain. Representative GREAT enrichment results were indicated on the right, values in parentheses indicated the $-\log_{10}(P\text{-value})$ of the related GO term.

Supplementary Fig. S13

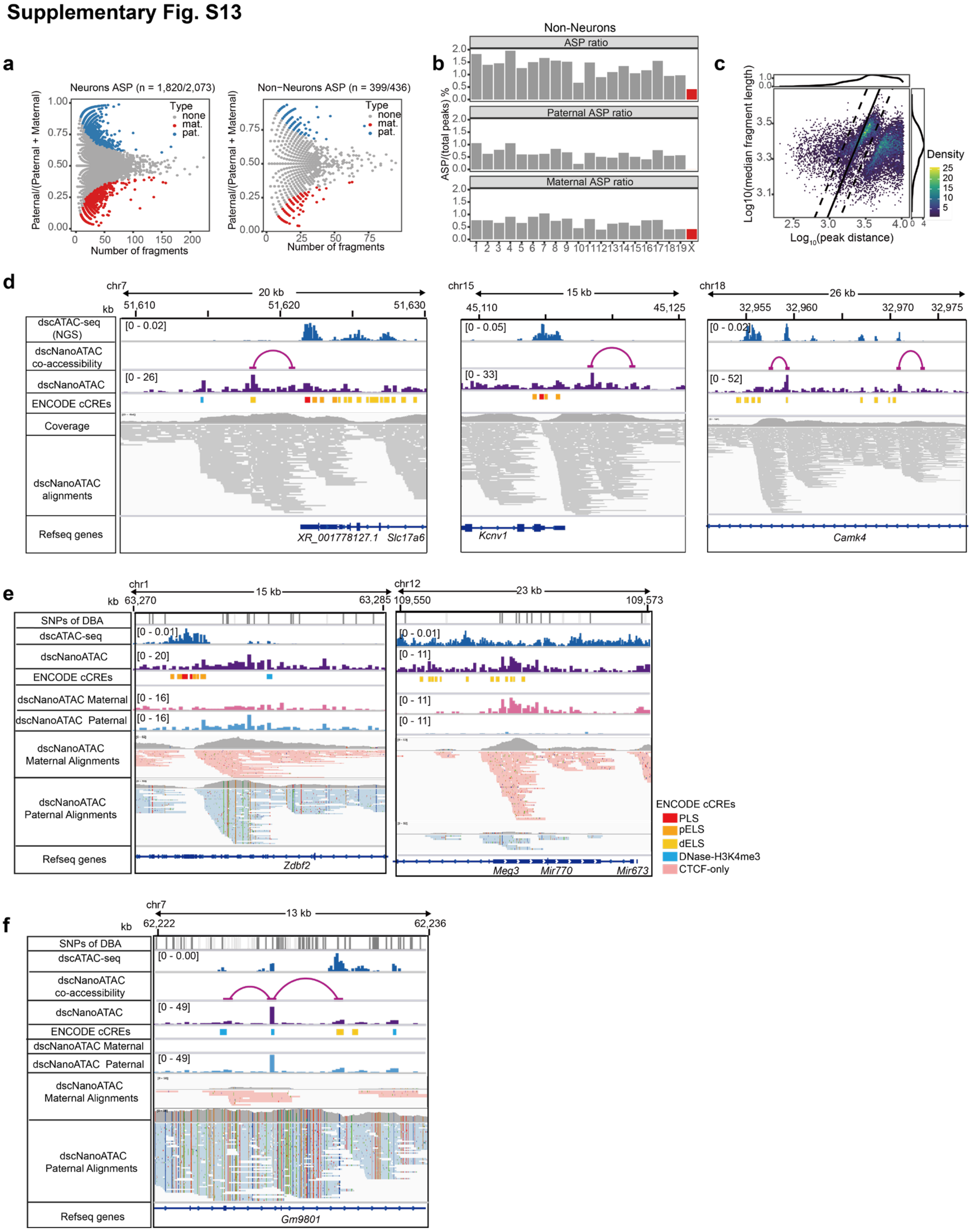

Supplementary Fig. S13 Representative examples of co-accessibility and allele-specific peaks in the mouse brain using dscNanoATAC. a. The ratio between paternal fragments and the total number of haplotyped reads for each peak in neurons (right) and non-neurons (left) identified by dscNanoATAC. Statistically significant allele-specific peaks (ASPs) (FDR < 0.05) are highlighted in blue (paternal) and red (maternal), respectively. **b.** The distribution of ASPs in non-neurons across chromosomes. **c.** Scatter plots indicating the filtration of co-accessibility events. **d.** Genome browser view showing representative co-accessibility events in *Slc17a6, Kcnv1, and CamK4* gene loci. **e.** Genome browser view showing the allele-specific peak (ASP) within the genome imprint gene locus *Zdbf2* and *Meg3*. **f.** Genome browser view showing representative co-accessibility events in allele-specific peak (ASP) within the gene locus *Gm9801***.**

Supplementary Fig. S14

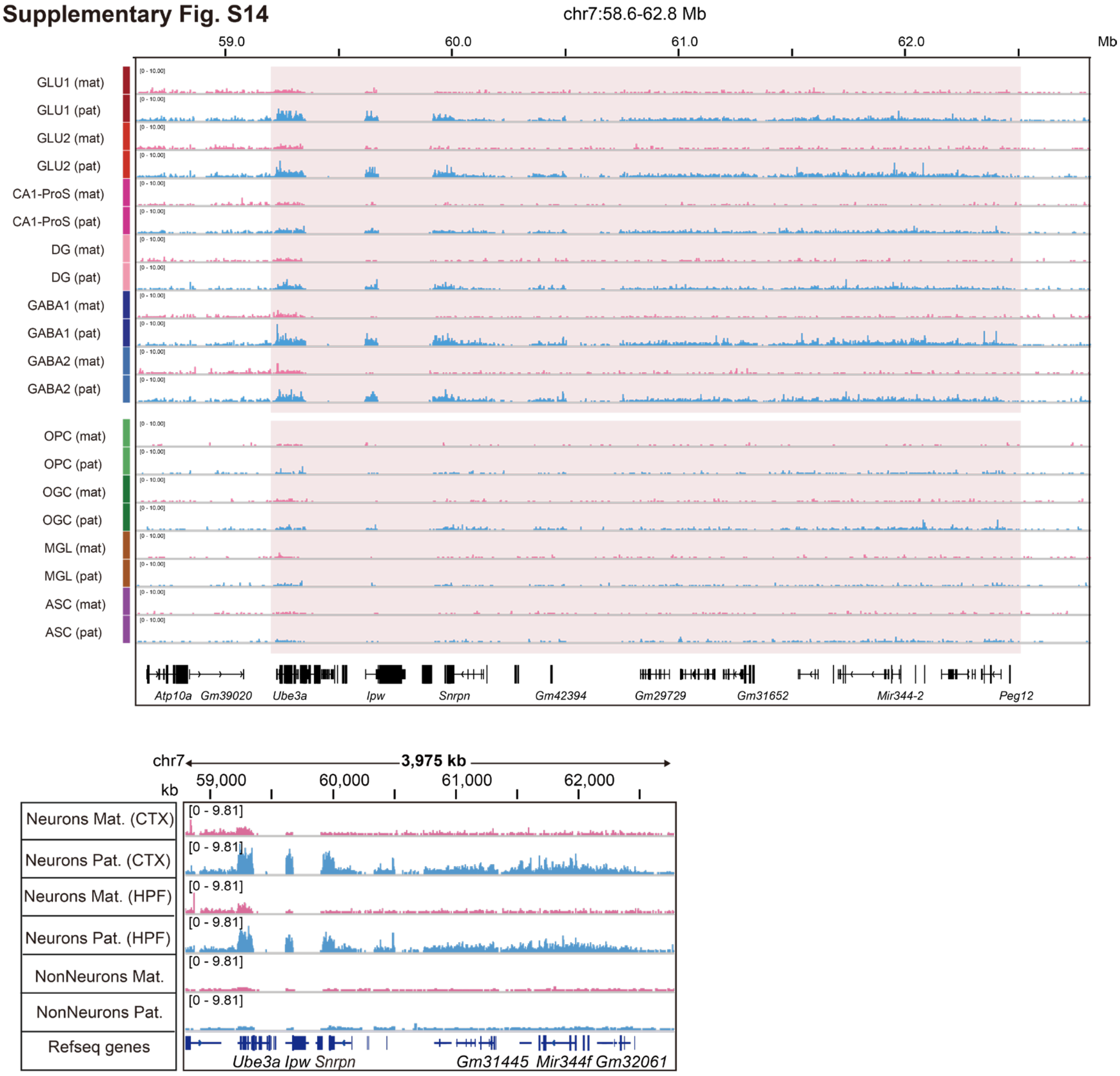

Supplementary Fig. S14 Allele-specific accessibility at the PWS/AS region of mouse brain. Genome browser view showing the allele-specific dscNanoATAC tracks at the PWS/AS region of the mouse brain, visualized by subtypes (top) and brain regions (CTX or HPF, bottom), respectively.

Supplementary Fig. S15

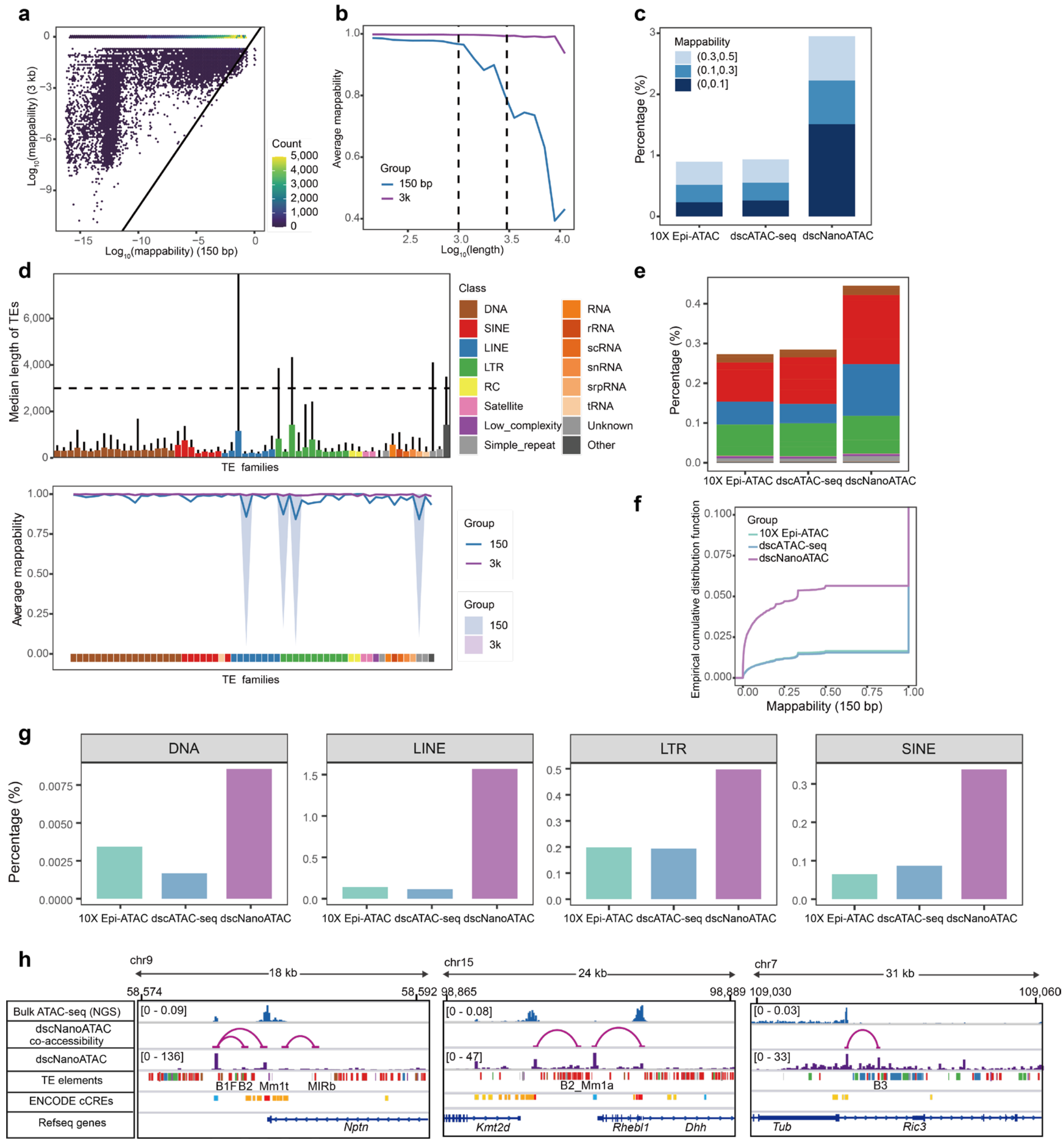

Supplementary Fig. S15 dscNanoATAC detected peaks in low-mappability regions in the mouse brain. a. The distribution of mappabilities (150 bp and 3 kb) on all copies of mouse repeat elements. **b.** The relationship between the length of repeat elements and their average mappabilities in 150 bp and 3 kb. **c.** Median length (top) and average mappability of TE families detected by 150 bp and 3 kb reads (bottom). **d.** The percentage of peaks located in low-mappability regions identified by 10X Epi-ATAC, dscATAC-seq (Lareau et al.)[12], and dscNanoATAC. **e.** The percentage of peaks located in low-mappability regions identified by 10X Epi-ATAC, dscATAC-seq (Lareau et al.), and dscNanoATAC in different TE families detected by 10X Epi-ATAC, dscATAC-seq (Lareau et al.), and dscNanoATAC. **f.** Empirical cumulative distribution of the mappability of peaks identified by 10X Epi-ATAC, dscATAC-seq (Lareau et al.), and dscNanoATAC, indicating the percentage of peaks located in low-mappability regions. **g.** The percentage of peaks located in repeat elements of DNA, LINE, LTR, and SINE classes detected by 10X Epi-ATAC, dscATAC-seq (Lareau et al.), and dscNanoATAC. **h.** Genome browser view showing representative regions containing accessible peaks at repeat elements, including the B2_Mm1t at the upstream of *Nptn*, B3 at the intron of *Ric3,*

and B3A upstream of *Rere*.

Supplementary Fig. S16

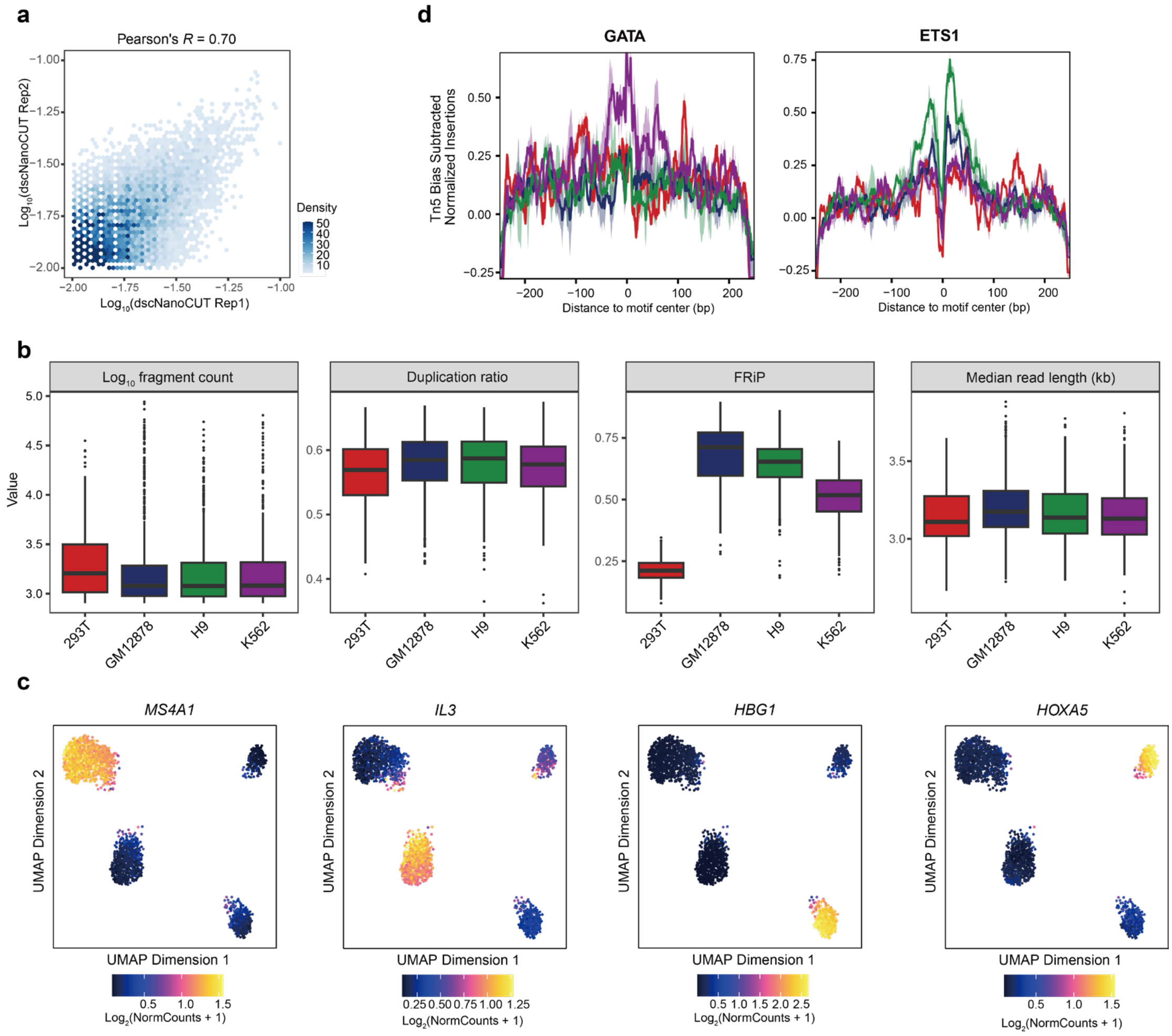

Supplementary Fig. S16 The quality control of dscNanoCUT data in four cell lines. a. The reproducibility of dscNanoCUT. Scatter plots illustrating the Pearson's correlation coefficients of the genome-wide H3K27ac signal between two independent biological replicates of K562. **b.** Quality control metrics of dscNanoCUT, including the number of fragments (Log_{10}-scaled), duplication ratio, FRiP (Fraction of Reads in Peaks), and median read length in four cell lines. **c.** H3K27ac gene scores visualized on UMAP embedding of four cell lines. **d**. The representative motif footprints of TFs, calculated from dscNanoCUT data.

Supplementary Fig. S17

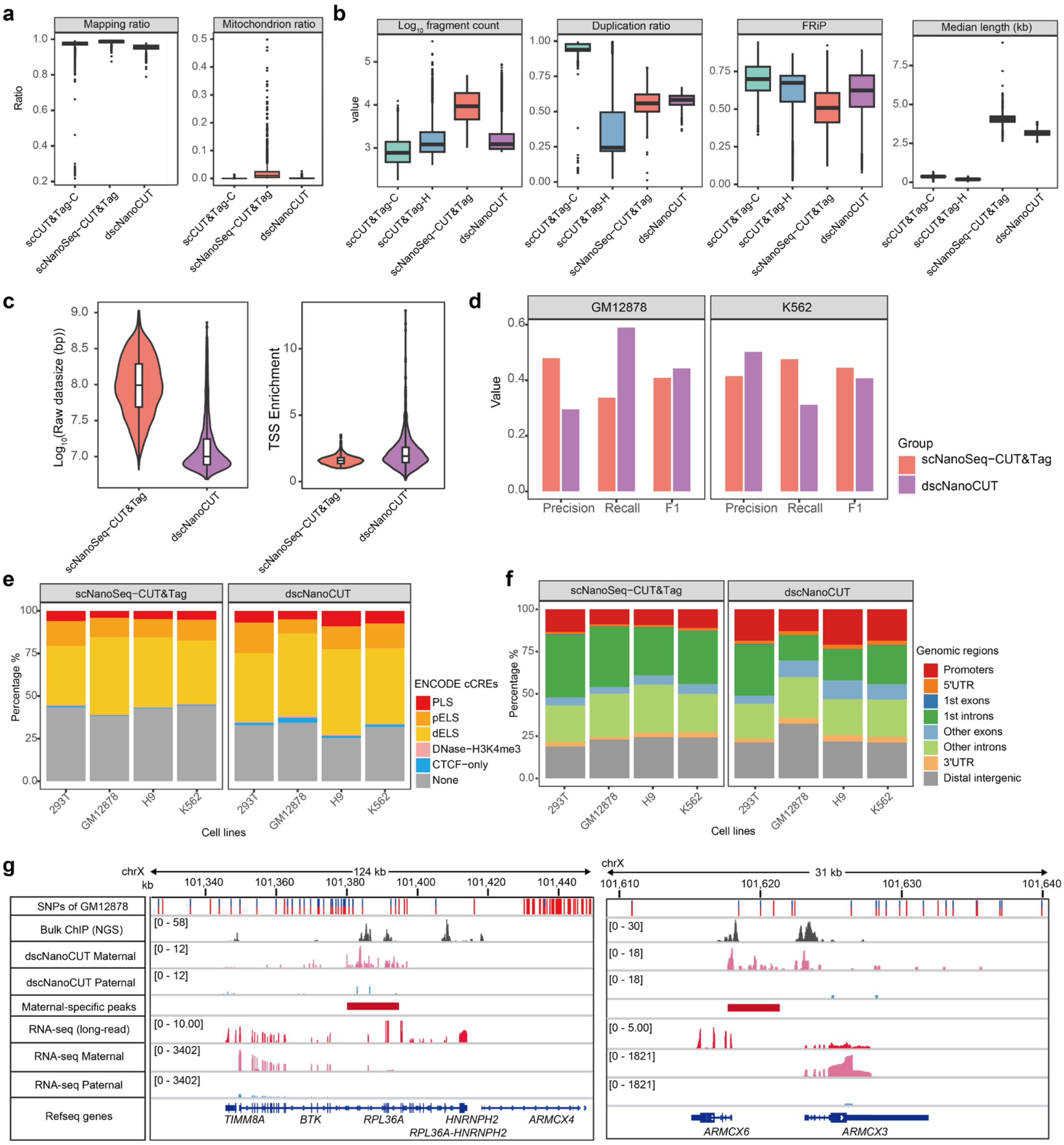

Supplementary Fig. S17 The quality control of dscNanoCUT data. a. Mapping ratio, mitochondrion identified by dscNanoCUT, scCUT&Tag-C (scCUT&Tag from Castelo-Branco lab) and scNanoSeq-CUT&Tag. **b.** Quality control metrics, including number of fragments (Log_{10}-scaled), duplication ratio, FRiP (Fraction of Reads in Peaks), and median read length identified by dscNanoCUT, scCUT&Tag-C (scCUT&Tag from Castelo-Branco lab)[9], scCUT&Tag-H (scCUT&Tag from Henikoff lab)[8], and scNanoSeq-CUT&Tag. **c.** The raw sequencing data size and TSS enrichment score between scNanoSeq-CUT&Tag and dscNanoCUT. **d.** The benchmarking results of the peaks identified from dscNanoCUT and scNanoSeq-CUT&Tag against the peaks of bulk ChIP-seq data. **e-f.** Distribution of H3K27ac peaks across candidate cis-regulatory elements (cCREs) (**e**) and gene regions (**f**) detected by dscNanoCUT and scNanoseq-CUT&Tag. **g.** Genome browser view showing representative regions of allele-specific peaks, including the known X chromosome inactive genes *BTK* and *ARMCX6*.

Supplementary Fig. S18

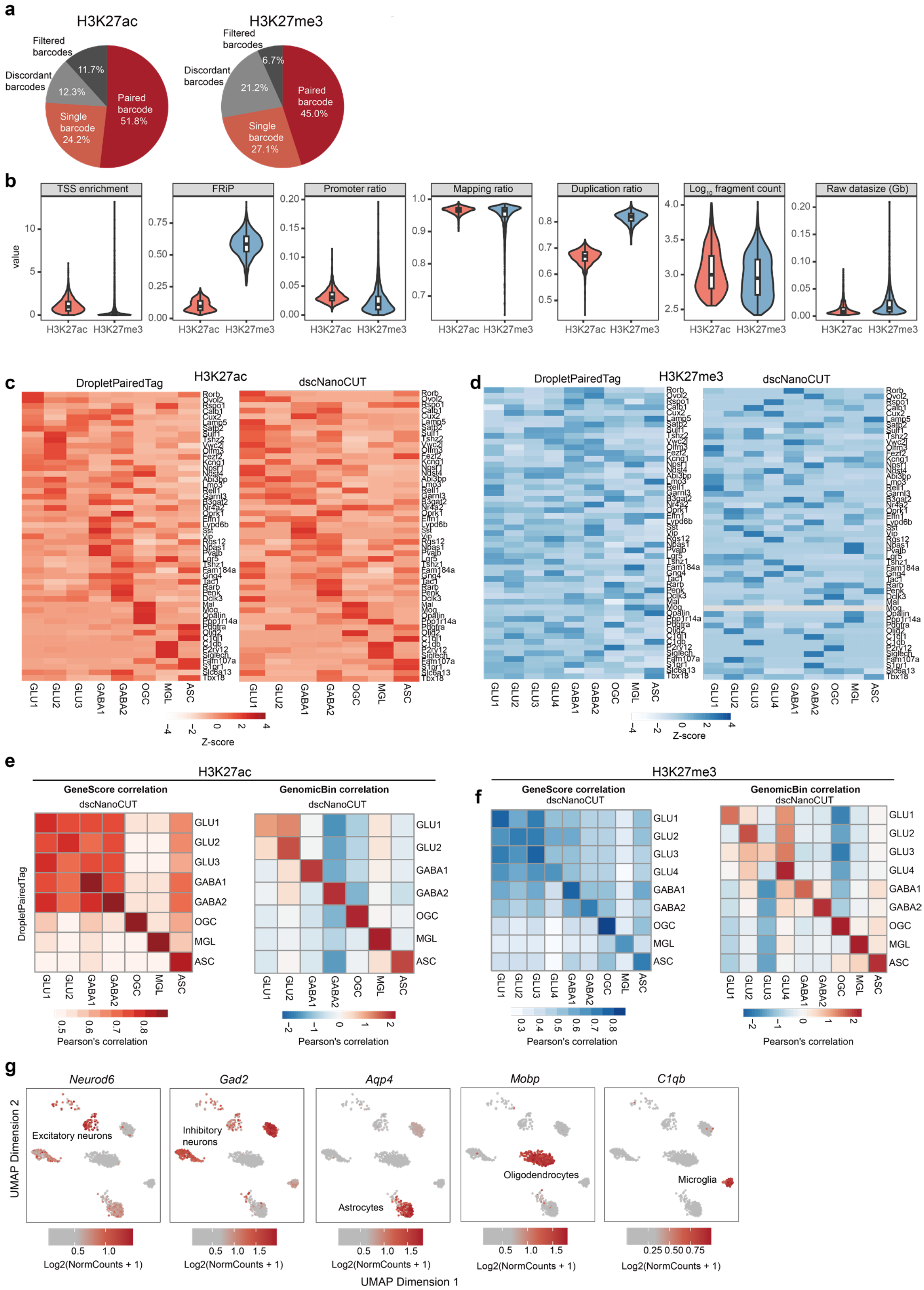

Supplementary Fig. S18 The quality control of dscNanoCUT data in the mouse brain. a. The demultiplexing summary of sequencing data for CTX in the mouse brain using dscNanoCUT. **b.** Quality control metrics, including TSS enrichment score, FRiP, promoter ratio, mapping ratio, duplication ratio, number of fragments (Log_{10}-scaled), and raw data size identified by dscNanoCUT using H3K27ac and H3K27me3. **c-d.** Heatmap showing the averaged Gene Scores of known marker

genes in each cell type in dropletPairedTag and dscNanoCUT using H3K27ac (**c**) and H3K27me3 (**d**). **e-f.** The gene score correlation between dropletPairedTag and dscNanoCUT using H3K27ac (**e**) and H3K27me3 (**f**). **g.** Gene scores of marker genes in each cluster are visualized on the UMAP embedding.

Supplementary Fig. S19

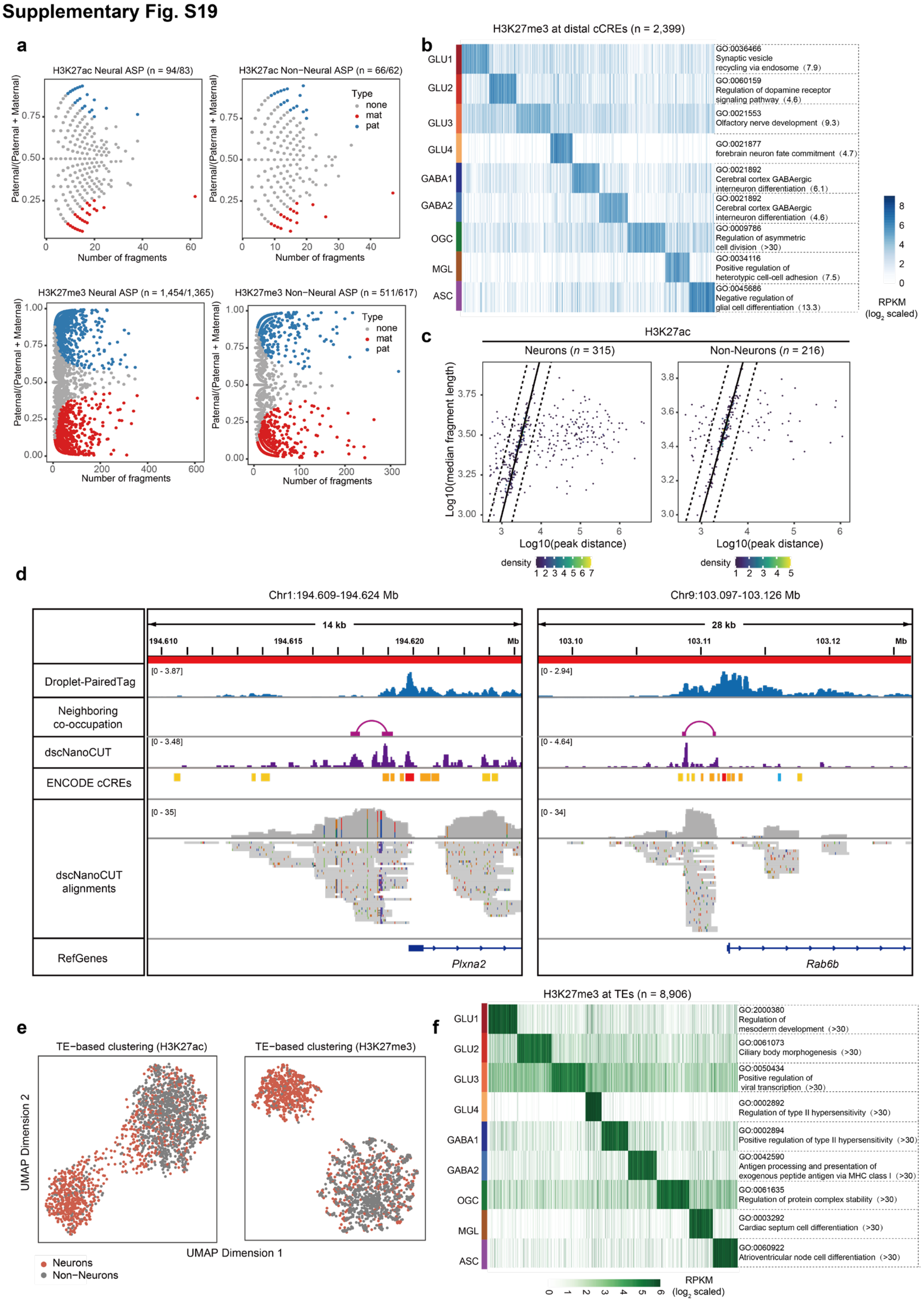

Supplementary Fig. S19 Allele-specific peaks and cCREs in mouse brain using dscNanoCUT.

a. The ratio between paternal fragments and the total number of haplotyped reads for each peak in neurons (right) and non-neurons (left) identified by dscNanoCUT using H3K27ac (up) and H3K27me3 (below). Statistically significant allele-specific peaks (ASPs) (FDR < 0.05) are highlighted in blue (paternal) and red (maternal), respectively. **b.** Distal cCRE modules of each cell type in the mouse brain using H3K27me3 modification. Representative GREAT enrichment results were indicated on the right, values in parentheses indicated the $-\log_{10}(P\text{-value})$ of the related GO term. **c**. identification of H3K27ac co-occupation events in neurons and non-neurons of the mouse cerebral cortex. **d**. Genome browser view of representative H3K27ac co-occupation events at *Plxna2* and *Rab6b* locus. **e.** UMAP showing the TE-based clustering of neurons and non-neurons using H3K27ac (left) and H3K27me3 (right). **f.** TE cCRE modules of each cell type in the mouse brain using H3K27me3 modification. Representative GREAT enrichment results were indicated on the right, values in parentheses indicated the $-\log_{10}(P\text{-value})$ of the related GO term.

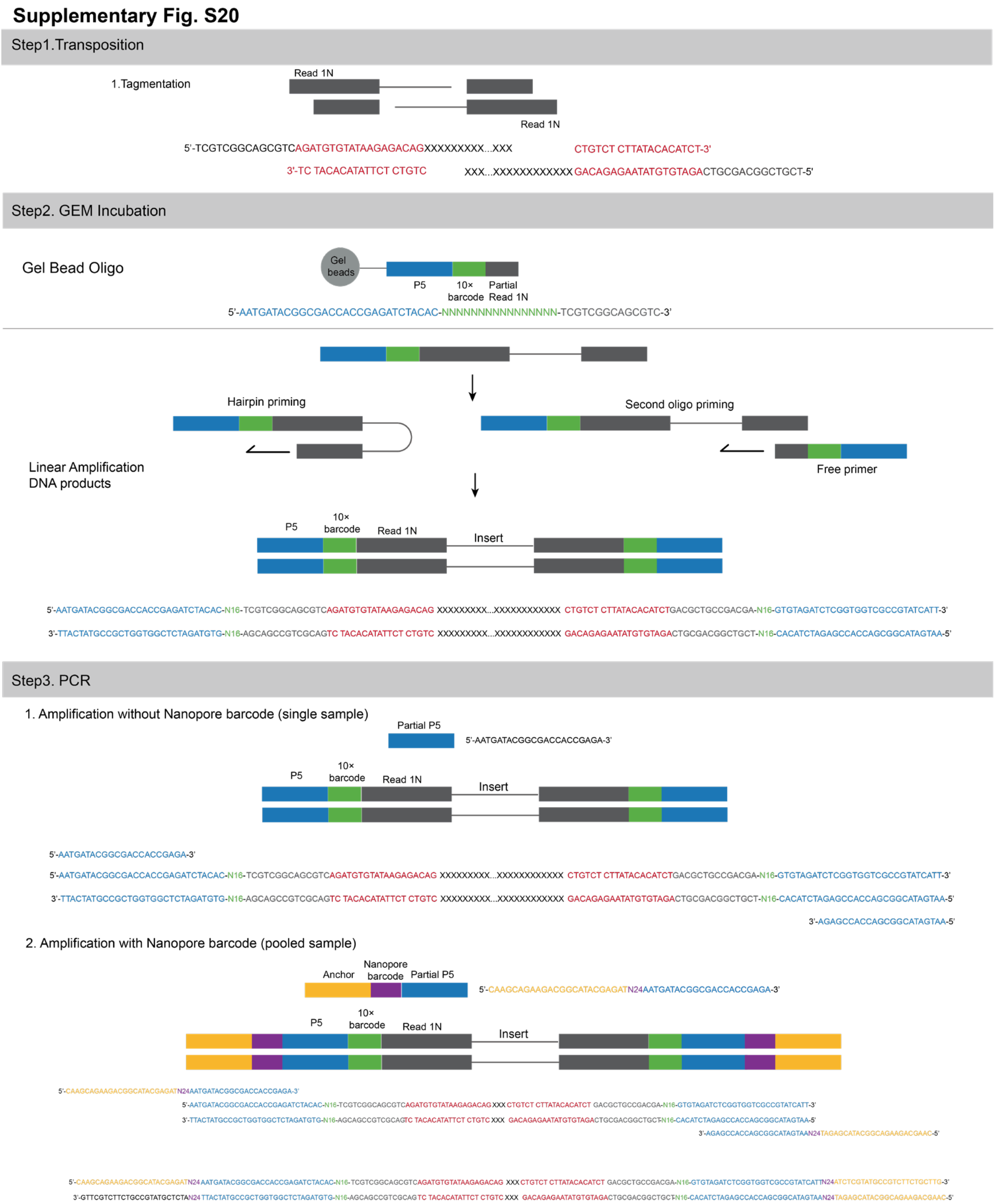

Supplementary Fig. S20 dscNanoATAC design on 10X Chromium platform. The genome was firstly transposed by mono-adapter Tn5 containing Read1N, then captured by bead oligos in the microfluidic platform. In extension and pre-amplification step, the 3' end is primed by 'hairpin priming' or 'second priming'. After that, product was amplified by a single primer containing Partial P5 and sequenced using Nanopore.

Supplementary Tables

Supplementary Table S1. Metadata of dscNanoATAC and dscNanoCUT

Supplementary Table S2. Allele-specific accessible peaks in GM12878 identified by dscNanoATAC

Supplementary Table S3. Neighboring co-accessibility in GM12878 identified by dscNanoATAC

Supplementary Table S4. GM12878 accessible peaks located in low-mappability regions

Supplementary Table S5. dscNanoATAC cCRE modules in the mouse brain

Supplementary Table S6. Allele-specific accessible peaks of the mouse brain

Supplementary Table S7. Neighboring co-accessibility in the mouse brain

Supplementary Table S8. Accessible peaks of the mouse brain located in low-mappability regions

Supplementary Table S9. Allele-specific H3K27ac peaks in GM12878 identified by dscNanoCUT

Supplementary Table S10. dscNanoCUT cCRE modules in the mouse brain

Supplementary Table S11. TE copies with H3K27ac or H3K27me3 modifications in the mouse brain

Supplementary Table S12. Primer information of dscNanoATAC and dscNanoCUT
